# Unusual evolution of tree frog populations in the Chernobyl exclusion zone

**DOI:** 10.1111/eva.13282

**Published:** 2022-01-26

**Authors:** Clément Car, André Gilles, Olivier Armant, Pablo Burraco, Karine Beaugelin‐Seiller, Sergey Gashchak, Virginie Camilleri, Isabelle Cavalié, Patrick Laloi, Christelle Adam‐Guillermin, Germán Orizaola, Jean‐Marc Bonzom

**Affiliations:** ^1^ Institut de Radioprotection et de Sûreté Nucléaire (IRSN) PSE‐ENV/SRTE/LECO Cadarache France; ^2^ UMR RECOVER INRAE Aix‐Marseille Université, Centre Saint‐Charles Marseille France; ^3^ Animal Ecology Department of Ecology and Genetics Evolutionary Biology Centre Uppsala University Uppsala Sweden; ^4^ Institute of Biodiversity, Animal Health and Comparative Medicine College of Medical, Veterinary and Life Sciences University of Glasgow Glasgow UK; ^5^ Chornobyl Center for Nuclear Safety Radioactive Waste and Radioecology Slavutych Ukraine; ^6^ Institut de Radioprotection et de Sûreté Nucléaire (IRSN) PSE‐SANTE/SDOS/LMDN Cadarache France; ^7^ IMIB‐Biodiversity Research Institute (Univ. Oviedo‐CSIC‐Princip. Asturias) Universidad de Oviedo Mieres‐Asturias Spain; ^8^ Department Biology Organisms and Systems Zoology Unit University of Oviedo Oviedo‐Asturias Spain

**Keywords:** Chernobyl, evolutionary ecotoxicology, genetic diversity, *Hyla orientalis*, ionizing radiation, mitochondrial mutations

## Abstract

Despite the ubiquity of pollutants in the environment, their long‐term ecological consequences are not always clear and still poorly studied. This is the case concerning the radioactive contamination of the environment following the major nuclear accident at the Chernobyl nuclear power plant. Notwithstanding the implications of evolutionary processes on the population status, few studies concern the evolution of organisms chronically exposed to ionizing radiation in the Chernobyl exclusion zone. Here, we examined genetic markers for 19 populations of Eastern tree frog (*Hyla orientalis*) sampled in the Chernobyl region about thirty years after the nuclear power plant accident to investigate microevolutionary processes ongoing in local populations. Genetic diversity estimated from nuclear and mitochondrial markers showed an absence of genetic erosion and higher mitochondrial diversity in tree frogs from the Chernobyl exclusion zone compared to other European populations. Moreover, the study of haplotype network permitted us to decipher the presence of an independent recent evolutionary history of Chernobyl exclusion zone's Eastern tree frogs caused by an elevated mutation rate compared to other European populations. By fitting to our data a model of haplotype network evolution, we suspected that Eastern tree frog populations in the Chernobyl exclusion zone have a high mitochondrial mutation rate and small effective population sizes. These data suggest that Eastern tree frog populations might offset the impact of deleterious mutations because of their large clutch size, but also question the long‐term impact of ionizing radiation on the status of other species living in the Chernobyl exclusion zone.

## 1 INTRODUCTION

The loss of biodiversity during the past 50 years is unprecedented in human history. Pollution, as part of the major drivers of biodiversity loss (namely habitat and climate change, pollution, overexploitation of natural resources, and invasive species), has severely altered many ecosystems (Brondizio et al., 2019). Among the large diversity of pollutants, radioactive contamination caused by human activities, and the associated risks for ecosystems and humans, are the subject of broad societal and scientific concern (Beresford & Copplestone, 2011). This is particularly true in the case of major nuclear accident such as the one occurred at the Chernobyl nuclear power plant (NPP) on April 1986 (Imanaka et al., 2015; Steinhauser et al., 2014). Although the short‐term adverse effects of high ionizing radiation doses on wildlife following this accident are not questioned (Alexakhin et al., 2006; Geras’kin et al., 2008; Møller & Mousseau, 2006), there are still many unknowns and controversies on the long‐term ecological consequences of these radioactive releases (Beresford, Horemans et al., 2020; Bréchignac & Paquet, 2013; Morgan & Bair, 2013; Mothersill & Seymour, 2013).

One of the biggest challenges for an accurate estimation of the impact of chronic pollution on ecosystems is to understand, quantify, and predict its effects not only at individual, but also at the population level of biological organization (Bickham, 2011; Medina et al., 2007; Theodorakis, 2001). Understanding the impact of pollutants on populations allows to investigate evolutionary processes that may affect population status and their capacity to persist in the future. Several studies in the Chernobyl area have estimated the abundance and interspecific diversity of wildlife after the accident (Bezrukov et al., 2015; Chapon et al., 2012; Deryabina et al., 2015; Lecomte‐Pradines et al., 2014; Gashchak et al., 2017; Gashchak et al., 2016; Møller & Mousseau, 2009, 2013, 2018; Møller et al., 2013; Morelli et al., 2018; Murphy et al., 2011; Schlichting et al., 2019; Shkvyria & Vishnevskiy, 2012; Zaitsev et al., 2014). However, these studies have provided inconclusive, and often divergent results, dependent on the sampling design (e.g., for mammals; Deryabina et al., 2015; Møller & Mousseau, 2013; Webster et al., 2016). In addition, studies investigating the evolution of wildlife in Chernobyl area are scarce and have not provided solid conclusions (Arnaise et al., 2020; Møller & Mousseau, 2016). In order to increase our understanding on the impact of ionizing radiation on wildlife in the Chernobyl area, we must examine intraspecific genetic variations. Examining genetic variations within and between populations may allow scientists to estimate differences in the intensity of possible evolutionary processes occurring in wildlife populations (Bickham et al., 2000; Giska et al., 2015; Straalen & Timmermans, 2002; Ungherese et al., 2010). Evolutionary processes (mutation, migration, genetic drift, selection) must be understood as the mechanisms that modify genetic variations within populations. Genetic diversity indices, in particular, can be highly informative from an ecological perspective since changes in genetic diversity can affect the capacity of populations to cope with environmental change (Fasola et al., 2015; Hughes et al., 2008; Luquet et al., 2011; Millette et al., 2019; Ribeiro & Lopes, 2013).

Populations exposed to pollutants often experience genetic erosion (Straalen & Timmermans, 2002). Two processes can be at the origin of this decreased diversity: a directional selective pressure which can be driven by the modification of the environment (De Wolf et al., 2004; Ungherese et al., 2010), and/or a demographic bottleneck involving the fixation of polymorphic alleles with neutral drift (Hughes et al., 2008; Murdoch & Hebert, 1994; Ribeiro et al., 2012; Wang et al., 2019). Numerous population genetic studies carried out in the Chernobyl area have been conducted on the bank vole, *Myodes glareolus*, and showed increased genetic diversity in highly radio‐contaminated areas (Baker et al., 2001, 2017; Matson et al., 2000; Meeks et al., 2007, 2009; Wickliffe et al., 2006). There are two explanations for this observation that are not mutually exclusive. First, exposure to radioactive pollution can lead to an increased mutation rate (Baker et al., 2017; Dubrova, 2003; Ellegren et al., 1997; Møller & Mousseau, 2015), which can partially offset the genetic diversity loss caused by population bottlenecks. Alternatively, the Chernobyl exclusion zone (CEZ)—which is an area established soon after the Chernobyl nuclear disaster where human population was evacuated (Bondarkov et al., 2011)—could act as an ecological sink (Dias, 1996; Matson et al., 2006; Møller et al., 2006; Pulliam, 1988; Theodorakis et al., 2001): a demographic deficit caused by the polluted habitat (mortality > natality) could lead to immigration to these habitats, and *in fine* to an increase in genetic diversity (Kesäniemi et al., 2018; Meeks et al., 2007).

Here, we examine the relationship between radionuclide contamination in the CEZ and the genetic pattern of populations in a lissamphibian species, the Eastern tree frog (*Hyla orientalis)* (Stöck et al., 2012) Bedriaga 1890 (Anura, Hylidae). The phylogeography of this species is well understood, which allows the examination of Chernobyl populations in the context of the general evolutionary history of the species (Dufresnes et al., 2016). In addition, the Eastern tree frog may be significantly exposed to ionizing radiation in both aquatic and terrestrial environments at susceptible stages of its development, especially during the metamorphosis and during its hibernation in the contaminated soil (Giraudeau et al., 2018; Stark et al., 2015).

**FIGURE 1:**
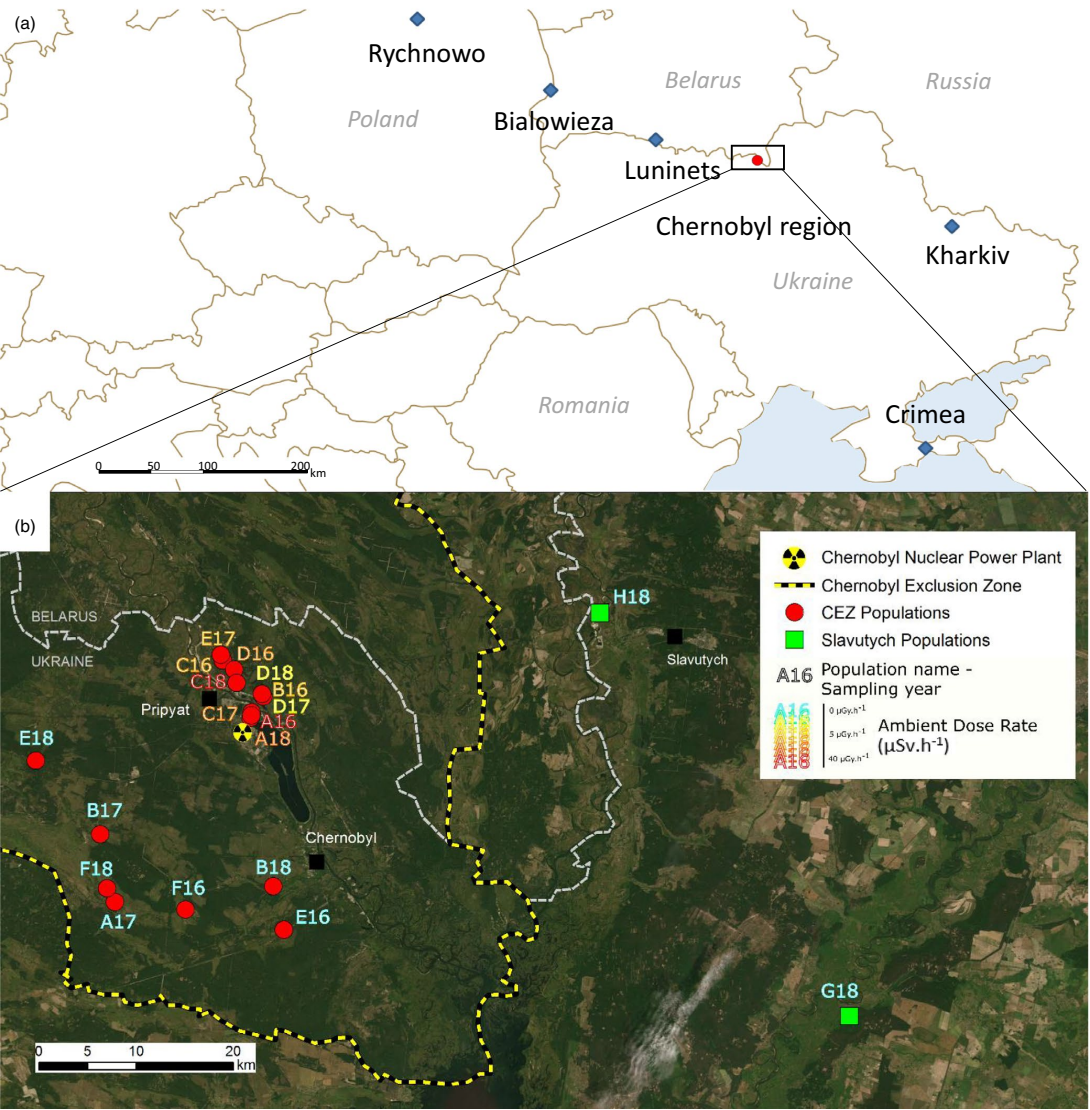
(a) Location of European populations of Eastern tree frogs outside the Chernobyl region sampled by Dufresnes et al. (2016) (blue diamonds) and the 19 populations sampled at the Chernobyl region (red circles). (b) Map of the Chernobyl region and location of the 19 populations sampled in 2016, 2017, 2018 in the CEZ and at Slavutych. The map was created with ArcGis v. 10.5. Source and service layer credits for satellite imagery: Esri, DigitalGlobe, GeoEye, Earthstar Geographics, CNES/Airbus DS, USDA, USGS, AeroGRID, IGN, and the GIS User Community

We studied population genetics from 19 populations of *H*. *orientalis* sampled about thirty years after the Chernobyl NPP accident at sites located across a wide range of radioactive contamination inside and outside the CEZ (Figure 1b). We used the cytochrome b coding gene as a mitochondrial marker and 21 nuclear microsatellites as nuclear markers. These markers differ in their mode of transmission, rate of evolution, and dynamics against environmental disturbances (Brown et al., 1979; Harrison, 1989; Selkoe & Toonen, 2006). Genetic diversity of populations from the CEZ was compared to that of populations up to 40 km distant from the CEZ (Slavutych), as well as to five other European populations belonging to the same clade (Dufresnes et al., 2016) (Figure 1a). Finally, we studied the mitochondrial haplotype network and simulated their networks over 10 and 15 generations to estimate the population parameters of frogs living in the CEZ since the accident.

## 2 MATERIALS AND METHODS

### 2.1 Fieldwork, capture, and preparation of the samples

In May and June 2016, 2017, and 2018 during the breeding season, we collected a total of 216 *H*. *orientalis* individuals in 17 populations in wetlands located inside the CEZ and 2 outside the CEZ, that is, in the Slavutych region (Figure 1b). For simplicity, we use here “population” in the meaning of “population sample.” These sites cover a gradient of ambient dose rates that was measured using a hand‐held radiometer (MKS‐AT6130, ATOMTEX). The mean (±*SD*) ambient radiation dose rate varied from 0.044 to 32.4 µSv/h. After capture, individuals were kept in individual boxes with a perforated cover and 2 cm of water until the next morning when they were euthanized and dissected to sample tibia muscle. Collected tissue was quickly frozen at −196°C, transported to IRSN labs in Cadarache (France), and stored at −80°C until DNA extraction. All animals were collected under the permit of Ministry of Ecology and Natural Resources of Ukraine (No. 517, 21.04.2016). The geographic distances separating each pairwise combination of frog populations were estimated with ArcGIS and a UTM projection.

### 2.2 Population‐averaged dose rate calculation

The approach for population‐averaged dose rate reconstruction was based on Giraudeau et al. (2018) (See Appendix S1: Note 2 for details). The two main differences compared with the protocol carried out by Giraudeau et al. (2018) are the radionuclides and the scenarios under consideration (Figure S4) because of the characteristics of the CEZ compared to the Fukushima situation. To summarize, soil activities (in Bq/kg) were extracted following Gashchak et al. (2010) from a spatial database using a geometric mean over a 400 m radius area centered on each population location and using a time correction, and water activities were calculated using soil activities and distribution coefficients estimated for the Glubokoye lake (Matsunaga et al., 1998). In addition, radioactivity concentrations (in Bq/kg) were estimated for each individual in femur bones for ^90^Sr, and in leg muscle for ^137^Cs in the IRL‐SSRI Laboratory (Slavutych, Ukraine), and then reconstructed for the total frog knowing the total frog mass and the relative mass of bones (10%) and muscles (69%) (Barnett et al., 2009). A Canberra‐Packard gamma‐spectrometer with a high purity germanium (HPGe) detector (GC 3019) was used for measuring ^137^Cs activity concentrations and a Beta‐spectrometer EXPRESS‐01 was used for measuring ^90^Sr activity concentrations. For a more detailed description of radioactivity measurement methods, see Beresford, Barnett et al. (2020). Then, dose coefficients (DCs) were calculated based on frog morphometry for internal exposure and four scenarios of external exposure using EDEN software (Beaugelin‐Seiller et al., 2006). DCs convert environmental radionuclide activity (Bq/kg soil, Bq/L water) into dose rate (µGy/h) for the frog, and are specific for each radionuclide/scenario/organism combination. The total dose rate (in µGy/h) was calculated for each frog combining related dose coefficients and activities. Average total dose rates (ATDRs) were obtained by averaging total dose rate of sampled individuals for each population (Figures S5 and S6). Only the radioactivity concentrations of ^90^Sr and ^137^Cs in frogs was measured, but the contribution to the total dose rate of other less abundant radionuclides (^241^Am, ^238^Pu, ^239^Pu) was estimated to contribute, on average, less than a quarter of the total dose rate (see Tables S9–S11 and Figure S7). Thus, the total dose rate we assessed could be underestimated by ~20 to 25% as radionuclides other than ^137^Cs and ^90^Sr were not included in the dose reconstruction. Nevertheless, in the CEZ soil radioactivity concentrations of ^90^Sr and ^137^Cs are correlated to the radioactivity of other less abundant radionuclides (Bonzom et al., 2016), as well as in the body of organisms such as small mammals (Beresford et al., 2008), thus our ATDR descriptor based on ^90^Sr and ^137^Cs is reliable for statistical tests.

### 2.3 DNA extraction, sequencing, and genotyping

DNA was extracted from tibia muscle using DNeasy Blood and Tissue Kit (Qiagen, Valencia, CA) following the manufacturer's protocol. After the estimation of nucleotide concentration with a spectrophotometric measurement and an electrophoresis quality check, a 957 bp fragment of mitochondrial DNA, cytochrome b, and 21 nuclear microsatellites were studied (see Appendix S1: Note 1 for details). Mitochondrial and nuclear markers were used simultaneously in order to compare their different properties. To sequence the cytochrome b, a PCR amplification was performed using Hyla‐L0 and Hyla‐H1046 primers (Dufresnes et al., 2016; Stöck et al., 2008). For each amplification session, a negative control was made using 3 µL of water instead of extracted DNA, and an electrophoresis was done to control the proper functioning of the amplification. PCR products were sequenced in both directions using Sanger sequencing (Eurofins, sequencing platform Cochin, France). The quality was checked using ab1 files. Sequences were aligned with MUSCLE program and corrected with MEGA (Kumar et al., 2016). In some cases, for the same position, an individual showed two different nucleotides. The mtDNA being haploid, it can be interpreted as a heteroplasmy (Hauswirth & Laipis, 1982; i.e., the presence of multiple mtDNA haplotypes in an individual). For each of these individuals, the two haplotypes were considered. Four multiplex amplifications were then performed for the 21 microsatellite markers (Dufresnes et al., 2014; Table S8). Formamide and a Size Standard were added to the PCR products and the whole was then genotyped with an ABI 3130 automated DNA sequencer (Applied Biosystems). Alleles were scored and genotyping was performed with GENE MAPPER 3.7 software (Chatterji & Pachter, 2006). A second amplification and genotyping were carried out on 4 individuals in order to check the replicability of the method.

### 2.4 Genetic analyses, mtDNA and nDNA

A quantitative analysis of population genetics was performed for the two types of markers. In order to avoid sample size artifacts, only populations with sample sizes greater than 6 individuals were used to describe the genetic diversity. Because sample sizes were still nonhomogeneous between populations, a rarefaction technique was performed for cytochrome b to calculate haplotypic richness (nrH) and private estimated haplotype number (npH) using hp‐rare (Kalinowski, 2005). The haplotype diversity (h) (Nei, 1987), nucleotide diversity (*π*) (Tajima, 1983) and three estimators of *θ* index (*θs*, *θk*, *θπ*) (Ewens, 1972; Tajima, 1983; Watterson, 1975) were calculated for the cytochrome b using ARLEQUIN (Excoffier et al., 2005). To describe how the mitochondrial genetic variation is structured temporally and geographically, Analysis of Molecular Variance (AMOVA) (Excoffier, 2004) and calculation of a differentiation index—pairwise *F*_st_—were performed using ARLEQUIN. Two AMOVA were performed using three year groups (2016, 2017, 2018), and three geographical areas corresponding to three groups of populations in the Chernobyl region: one in the North close to the NPP, one South of the exclusion zone, and one including the Slavutych populations (Figure 4c). For microsatellites markers, we estimated the observed heterozygosity (Ho), the estimated heterozygosity under Hardy–Weinberg assumptions (He), the genetic diversity (Hs), the allelic richness (AR), and the private allelic richness (PA) using GENETIX (Belkhir et al., 2004), ADZE (Szpiech et al., 2008), and *F*_stat_ (Goudet, 1995). Pairwise *F*_st_ for microsatellites were calculated using *F*_stat_. The absolute value of the lowest *F*_st_ for the mitochondrial and nuclear markers was added to every pairwise *F*_st_ in order to get only positive pairwise *F*_st_. We calculated the ratio *F*_st_/(1–*F*_st_) in order to estimate genetic distance between populations, and represented these distances using Neighbor‐Joining trees with genetic distances estimated with MEGA (Kumar et al., 2016).

Following the first quantitative analysis, we then focused on the cytochrome b mitochondrial marker as it allowed us to study qualitatively haplotypes of all populations (i.e., CEZ, Slavutych, and populations outside Chernobyl region sampled by Dufresnes et al. (2016); see Figure 1a), and examined their genealogical links within a network of haplotypes. This approach is a good way to situate populations within an evolutionary context and explore more subtle evolutionary processes than with diversity indices only (Matson et al., 2006). The haplotypes were determined using DNAsp (Rozas et al., 2017), the haplotype network was calculated with the Median‐Joining method (Bandelt et al., 1999) and drawn using POPART (Leigh & Bryant, 2015).

### 2.5 Simulation of haplotype networks

Simulations of mitochondrial haplotype networks were conducted with a method close to the Approximate Bayesian Computation (Beaumont et al., 2002) (for details on protocol see Appendix S1: Note 3). Simulations of the haplotype evolution for 10 and 15 generations (assuming one generation every three versus two years; Altunisik & Özdemir, 2013; Özdemir et al., 2012) in a unique population were conducted in R and Pegas library (Paradis, 2010) using different parameters: (i) the founder population size (*N_0_*) corresponding to specimens able to reproduce after the accident, (ii) the frequencies of haplotypes in the founder population based on the current diversity observed for the Slavutych populations (H18 and G18), (iii) the population size for each year during the 10 or 15 generations (*N*_1−_ *_n_*), (iv) the nucleotide substitution rate (*µ*) and (v) the number of generations. A haplotype network was generated for the last generation for each data set obtained with a set of value for prior parameters.

A first simulation was run with classical wild frog population prior parameters (i.e., notably a fluctuating population size since the accident in the range of uniform distribution U(1000–5000) and a classical rate of nucleotide substitution in mitochondrial DNA for amphibians of 20.37 × 10^−9^ per nucleotide per generation; Lynch & Walsh, 2007), representing 1000 simulations for each modality combination, a total of 6000 simulations. Then, a second simulation was performed with different prior parameters calibrated from the first simulation results (i.e., notably smaller population sizes with three modalities of uniform distribution: U(50,100), U(100,200), and U(200,300) and high nucleotide substitution rates with six modalities: 0.005, 0.01, 0.02, 0.04, 0.06, 0.08 per haplotype per generation in an infinite site model), corresponding to 100 simulations for each modality combination, a total of 21,600 simulations (see Tables S12 and S13 for prior parameters details).

Each haplotype network was described by five statistics: the nucleotide diversity (*π*), the Tajima's D, the haplotype richness (*nrH*), the haplotype diversity (h), and the number of steps separating the ancestral haplotype to the most distant haplotype plus one. A Principal Component Analysis (PCA) was performed to compare simulated and observed descriptive statistics. The two first principal component axes were used to visualize both sets of descriptive statistics. A Ward hierarchical cluster analysis on Euclidean distance was used to select the 5th percentile of the simulated descriptive statistics closest to the observed descriptive statistics. The mean and median of the 5th percentile simulated descriptive statistics were calculated to estimate the posterior parameters *N_0_*, the appropriate haplotype frequencies for the founder population, *N*_1−_ *_n_*, and *µ*. Visualization of the haplotype network for prior, 10th, and 15th generation was done using TempNet (Prost & Anderson, 2011).

### 2.6 Statistical analysis

Nonparametric Wilcoxon signed rank tests were performed to compare genetic diversity indices, between CEZ populations and outside CEZ populations, because of their non‐normal distribution tested using Shapiro–Wilk test. Nonparametric Spearman rank tests were used to test the correlation between genetic diversity indices and the population‐averaged dose rate (ATDR). The correlation between two matrices, the genetic distance matrix obtained with the *F*_st_ linearization and the matrix of logarithm of geographical distance (in m) (“isolation by distance” hypothesis), was performed using a Mantel test (Mantel, 1967) with the vegan package (Oksanen et al., 2009). Because of a possible link between geography and radionuclide contamination (Kashparov et al., 2018), the part of pairwise population‐averaged dose rate differences between populations on the distance correlation was tested using a partial Mantel test. The significance of Mantel tests was estimated with 9999 permutations. All these tests were carried out on R version 3.6.1. (R Core Development Team, 2009).

To test the demographical expansion hypothesis with haplotype networks, three statistical tests were performed on DNAsp. The neutral assumption of the absence of deviation from the mutation‐drift equilibrium was tested using the Tajima's *D* (Tajima, 1989) and Fu's D* (Fu & Li, 1993). The distribution of pairwise differences between sequences was studied too, using the *R* ^2^ statistic (Ramos‐Onsins & Rozas, 2002).

## 3 RESULTS

### 3.1 Mitochondrial DNA heteroplasmies

In the Chernobyl region, we observed 20 substitutions composed of 19 transitions (12 C/T and 7 A/G) and 1 transversion (A/T) (Tables S1 and S2). We determined that haplotypes found were part of the clade D4 described for other areas of Europe (Dufresnes et al., 2016; Stöck et al., 2012) and characteristic of areas from the northern Black Sea shores to the Baltic Sea. In addition, 7 individuals were interpreted as case of heteroplasmy in the CEZ, while none were detected in the other European populations (Figure S1 and Table S3).

### 3.2 High mitochondrial genetic diversity for CEZ populations

**FIGURE 2:**
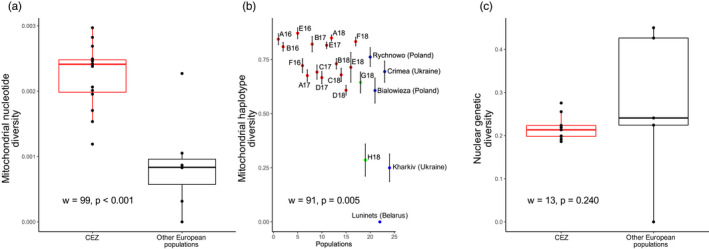
Comparison between genetic diversity estimates at the European level. (a) Boxplot of mitochondrial nucleotide diversity (i.e., the probability that two randomly chosen nucleotides of the cytochrome b at a homolog position are different; Nei, 1987; Tajima, 1983) for CEZ (red) and other European populations (black). Genetic diversity is higher at the CEZ than at other European populations (Mann–Whitney, *w* = 99, *p* = 0.0004). (b) Mitochondrial haplotype diversity estimates (i.e., the probability that two randomly chosen haplotypes of the cytochrome b are different (Nei, 1987)) ± standard error for CEZ (red), populations from Slavutych (green) and sampled by Dufresnes et al. (2016) (blue). Genetic diversity is higher at the CEZ than at other European populations (Mann–Whitney, *w* = 91, *p* = 0.005). (c) Boxplot of nuclear genetic diversity estimated on the 21 microsatellites markers (Nei, 1987) for CEZ (red) and other European populations (blue). There are no significant differences between the genetic diversity of CEZ and other European populations (Mann–Whitney, *w* = 13, *p* = 0.240)

Mitochondrial haplotype and nucleotide diversities of all populations from CEZ (*h* = 0.7308, *π* = 0.0024) were significantly higher (*W* = 91, *p* < 0.005) than those of other European populations (*h* = 0.6071, *π* = 0.0008) (Figure 2a and Table S4). In particular, for these other European populations, the lowest mitochondrial haplotype diversity was measured for the populations nearest to Chernobyl region, in Kharkiv, Ukraine (*h* = 0.2500) and Luninets, Belarus (*h* = 0) (Figures 1a and 2b). The two Slavutych populations present an intermediate mitochondrial haplotype diversity, as the H18 population had low genetic diversity (*h* = 0.2857), while the G18 population had a genetic diversity fairly close to the genetic diversity of the CEZ populations (*h* = 0.6444) (Figure 2b). Unlike mtDNA, all the indices of estimated nuclear genetic diversity, such as the genetic diversity within populations (Hs) (Figure 2c), showed no significant differences between the CEZ populations and the other European populations (Table S5).

**FIGURE 3:**
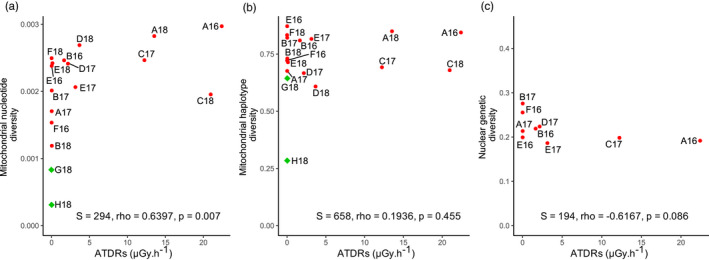
Correlation plots representing genetic diversity estimates on population‐averaged dose rate (ATDR) in µGy/h. Only populations of the Chernobyl region (i.e., CEZ (red dots) and Slavutych (green diamonds), Figure 1b) with sample size >7 individuals were compared. (a) Mitochondrial nucleotide diversity estimates (i.e., the probability that two randomly chosen nucleotides of the cytochrome b at a homolog position are different (Nei, 1987; Tajima, 1983)) on ATDR of the corresponding population. Nucleotide diversity is positively correlated to ATDR (*S* = 294, rho = 0.6397, *p* = 0.007). (b) Mitochondrial haplotype diversity estimates (i.e., the probability that two randomly chosen haplotypes of the cytochrome b are different (Nei, 1987)) on ATDR of the corresponding population. Haplotype diversity is not correlated to ATDR (*S* = 658, rho = 0.1936, *p* = 0.455). (c) Nuclear genetic diversity (Hs) was estimated on the 21 microsatellites markers (Nei, 1987) on ATDR. Genetic diversity is not correlated to ATDR (*S* = 194, rho = −0.6167, *p* = 0.086)

Only mitochondrial nucleotide diversity was significantly positively correlated to ATDRs (*S* = 294, rho = 0.640, *p* = 0.007) (Figure 3a and Table S6). In contrast, the correlation between mitochondrial haplotype diversity and ATDR was not significant (*S* = 658, rho = 0.193, *p* = 0.455; Figure 3b). Genetic diversity in nuclear microsatellites was not significantly correlated with ATDRs, although these parameters showed a nonsignificant negative correlation (*S* = 194, rho = −0.617, *p* = 0.086; Figure 3c, Figure S3). Only private allelic richness and ATDRs were significantly negatively correlated (*S* = 221.13, rho = −0.843, *p* = 0.004) (Table S7).

### 3.3 Local geographical structure of genetic variation

**FIGURE 4:**
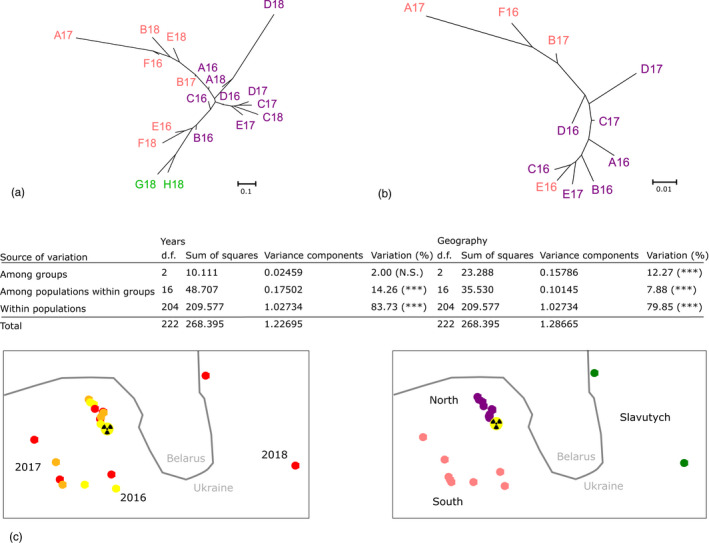
Genetic structure of the 19 populations of Eastern tree frogs from CEZ and Slavutych. Neighbor‐joining trees were constructed from genetic distances calculated as $F_{{\text{st}}_{\text{posi}}}/\left(1‐F_{{\text{st}}_{\text{posi}}}\right)$ with $F_{{\text{st}}_{\text{posi}}}$ equal to the addition of *F*_st_ and the absolute value of the lowest *F*_st_ in order to avoid negative values and respect proportionality of pairwise *F*_st_. (a) Neighbor‐Joining tree of CEZ (purple and pink) and Slavutych (green) populations from cytochrome b (mtDNA). (b) Neighbor‐Joining tree of CEZ populations (red) from microsatellites (nDNA). (c) AMOVA analysis conducted on Year and Geographical groups on mtDNA. Stars represent significance calculated from Arlequin with 1023 permutations (Excoffier et al., 2005) (***: sign <0.001). Year groups are 2016, 2017, 2018 (2016: yellow, 2017: orange, 2018: red) and geographical groups are north close to the Chernobyl Nuclear Power Plant (radiation warning symbol), south distant from the north and Slavutych (north: purple, south: pink, Slavutych: green)

Unlike the low differentiation estimated with nuclear microsatellites (−0.031 < *F*_st_ < 0.093), differentiation estimated from cytochrome b sequences was relatively high (−0.173 < *F*_st_ < 0.426). For the mitochondrial marker, the two populations with the highest genetic differentiation from other populations of the Chernobyl region were the South West A17 and the North D18 (0.072 < *F*_st_ < 0.426, Figure 4a). Slavutych populations were also highly genetically differentiated from CEZ populations (0.095 < *F*_st_ < 0.395). Genetic pairwise differentiations estimated on nuclear markers were similar to those estimated on mitochondrial markers, the most differentiated population being A17 (0.021 < *F*_st_ < 0.093, Figure 4b). Despite the absence of a complete similarity between geographical and genetic structures (Figures 1b and 4a,b), the genetically closest populations were, as expected, usually the geographically closest populations. This similarity was obvious when separating populations in Neighbor‐Joining (NJ) trees built for each sampling year (Figure S2).

In both AMOVA, the highest variance was observed within populations (83.75% and 79.85%). However, the inter‐group variance based on years was not significant (2.00%, *p* > 0.05), in opposition to the variance based on geographical regions (12.27%, *p* < 0.001).

Isolation by distance was significant for both nuclear (*r* = 0.4453, *p* = 0.005), and mitochondrial markers (*r* = 0.3461, *p* = 0.009). The correspondence between nuclear and mitochondrial genetic distances described from NJ trees was also significant (*r* = 0.6627, *p* = 0.003). The correlation between genetic distances and geographic distances could not be explained by the differences in ATDRs (mtDNA: *r* = 0.3474, sign = 0.007; nDNA: *r* = 0.4452, sign = 0.006).

### 3.4 Haplotype networks and CEZ‐independent evolutionary processes

**FIGURE 5:**
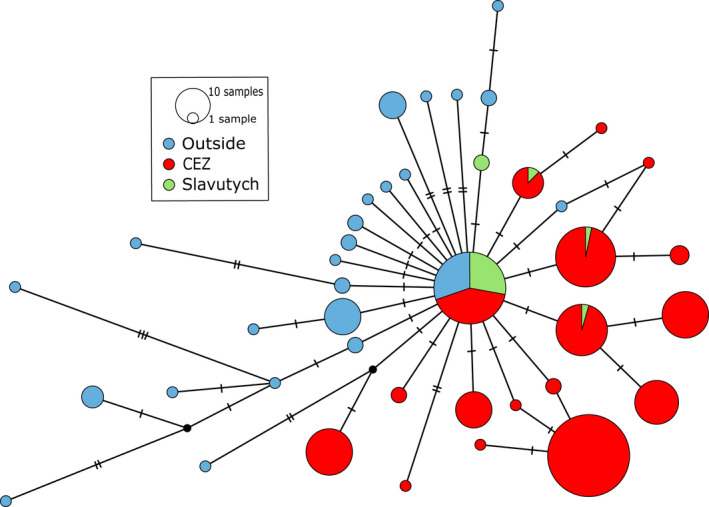
Haplotype network constructed for Eastern tree frog cytochrome b sequences from CEZ (red), Slavutych (green) populations, and European populations sampled by Dufresnes et al. (2016) (blue) using the Median‐Joining method (Bandelt et al., 1999) and POPART software (Leigh & Bryant, 2015). Circles representing haplotypes, their diameter is proportional to the number of individuals and the number of horizontal bars between haplotypes representing the number of nucleotides differing between haplotypes. The network structure can inform on the demographic status of populations: when the central haplotype is large compared to the surrounding haplotypes and lot of one step rare haplotypes surround this central haplotype (e.g., Slavutych and European populations), it is an indication of demographic expansion; if the central haplotype is not mainly represented and if there are a lot of two or three steps large haplotypes, it is an indication of a population at the equilibrium mutation/drift and this population is often formerly diversified (CEZ populations)

We identified a single haplotype common to all populations (CEZ, Slavutych area, and outside the Chernobyl region), the central haplotype (Figure 5). Because of the star‐like distribution of the haplotype network of populations outside the Chernobyl region (in blue, Figure 5) with respect to this central haplotype, we considered it as the ancestral haplotype.

We detected a discrepancy between the structure of the CEZ haplotype network and those of all other populations, since the population sampled in Slavutych segregated similarly to populations from other European areas analyzed by Dufresnes et al. (2016): the largest haplotype was the central haplotype, surrounded by many one substitution step rare haplotypes (Figure 5; green and blue). These populations outside the CEZ are in demographic expansion, as confirmed by the rejection of the equilibrium mutation/drift hypothesis (Tajima's *D* = −2.2180, *p* < 0.01; Fu and Li D* = −4.4028, *p* = 0.002; *R* ^2^ = 0.0289, *p* = 0.001). In contrast, the CEZ populations present a different pattern represented by haplotypes at one and two steps from the central haplotype, shared by many individuals (Figure 5, in red), and these populations are not in demographic expansion (Tajima's *D* = −0.5641, *p* = 0.332; Fu and Li D* = −1.4653, *p* = 0.089; *R* ^2^ = 0.0663, *p* = 0.357).

### 3.5 Small populations and elevated mutation rate in the CEZ

**FIGURE 6:**
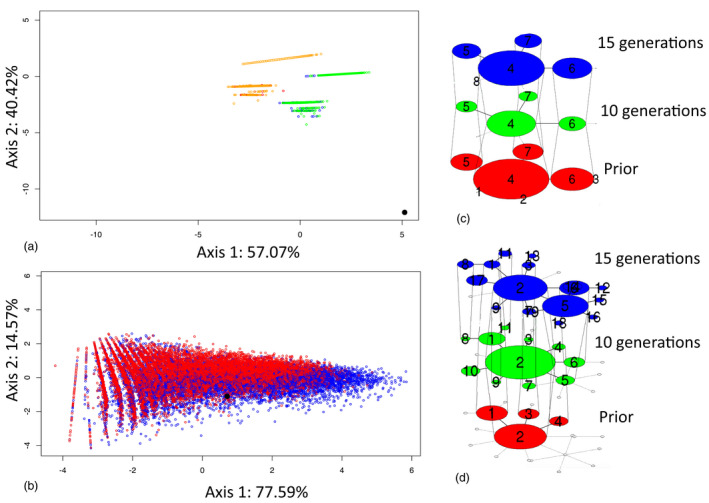
Mitochondrial haplotype network simulation. (a) Representation of observed (black circle) and simulated (colored open circles) data with a classical amphibian mitochondrial nucleotide substitution rate (20.37 × 10^−9^ per nucleotide per generation), a population size sampled in a uniform distribution U(1000–5000), and starting from the H18 (orange) or G18 (green) population haplotype frequencies on the two first axis of a PCA made on a set of haplotype network statistics. The observed data are not in the space of the simulated data. (b) Representation of observed (black circle) and simulated (colored circles) data with a high mitochondrial nucleotide substitution rate (0.005, 0.01, 0.02, 0.04, 0.06, 0.08) and small population size (sampled in a uniform distribution U(50,100), U(100,200), or U(200,300)) for 10 (red) and 15 (blue) generations on the two first axis of a PCA made on a set of haplotype network statistics. The observed data is in the space of the simulated data. (c) One example of haplotype network evolutionary scenario of a simulated population starting from G18 population haplotype frequencies as prior with a classical amphibian substitution rate and populations sizes in the range of uniform distribution U(1000–5000) (d) One example of haplotype network evolutionary scenario of a simulated population starting from G18 population haplotype frequencies as prior with a high substitution rate (0.04) and a maximal effective size of 100 (for prior (red) and 10 (green) and 15 (blue) generations after the Chernobyl nuclear power plant accident)

During the first simulation with classical wild frog population prior parameters, simulated haplotype networks did not match the observed one, the closest Euclidean distance being 11.13. The diversity of CEZ populations cannot thus be obtained with this first set of parameters (Figure 6a, c and Figure S8).

In contrast to the first simulation, PCA obtained from the second simulation displayed a match between the haplotype network statistics of the simulated and observed data. Indeed, the observed data was in the space of simulated data based on the two first principal components supporting around 90% of variance (Figure 6b). The closest distance was 0.52 and the median of descriptive statistics for the 5 percentile closest simulated values presented important similarity with observed values (Figure S9). Considering the posterior parameters estimated, the diversity of CEZ populations and the particular haplotype network (Figure 6d) for the studied mitochondrial marker can thus be obtained in 15 generations with a small population (N_max_ =100) and a high nucleotide substitution rate of 0.04 per haplotype per generation (Figure S10).

## 4 DISCUSSION

Several studies have shown that in the CEZ, where all human residents have been evacuated, large mammals in particular are reappearing, doubtless due to a decrease in human disturbance to wildlife (Deryabina et al., 2015; Gashchak et al., 2016, 2017; Shkvyria & Vishnevskiy, 2012). Conversely, other studies have shown a decrease in the abundance of some species in the CEZ (birds, Møller & Mousseau, 2007; insects, Møller & Mousseau, 2009; mammals, Møller & Mousseau, 2013). There is still no consensus about the long‐term consequences of the Chernobyl NPP accident, and the effects of exposure to ionizing radiation on population status remain controversial. To date, very few studies have focused on the evolutionary processes occurring in natural populations that underwent chronic exposure since the 1986 Chernobyl NPP accident. To the best of our knowledge, our study is the first in the Chernobyl region (i) investigating the evolutionary processes of CEZ populations, in comparison to the global European evolution of the closest lineage to which they belong, (ii) using both qualitative and quantitative mitochondrial genetic information and quantitative nuclear genetic information to estimate the best evolutionary scenario responsible of the observed pattern.

### 4.1 A higher mtDNA diversity in the CEZ driven by mutation process

In contrast to the expected genetic erosion induced by wildlife exposure to a pollutant (Straalen & Timmermans, 2002), our results did not show a genetic bottleneck of *H*. *orientalis* populations in the CEZ compared to the other European populations studied by Dufresnes et al. (2016). We found a higher mitochondrial genetic diversity for the populations in the CEZ, while similar nuclear genetic diversity was observed between CEZ populations and other European populations. These results on mitochondrial diversity agree with the increased mitochondrial genetic diversity observed in bank voles, *Myodes glareolus*, from the most contaminated areas of the CEZ (Baker et al., 2017). A higher diversity can be explained by two evolutionary processes: migrations from multiple distinct and distant populations, or a local higher mutation rate. Because of the discrepancy between nuclear and mitochondrial markers, the mutation process seems to be the best explanation for this higher diversity (Canestrelli et al., 2006; Toews & Brelsford, 2012).

Indeed, repair mechanisms in mtDNA are usually considered less effective than in nDNA (Kazak et al., 2012; Larsen et al., 2005) notably because of variations in replication mechanism (i.e., low fidelity of the DNA polymerase γ) and a higher number of genome replications per generation, especially during oocyte maturation (Allio et al., 2017). Thus, the emergence of a mutagenic factor in the environment can induce mutations on mtDNA without increasing nDNA mutations at the same rate. The same kind of difference between these two types of DNA has already been observed in subterranean waterlice (Saclier et al., 2020). When these species were exposed to natural radioactivity, the mutation rate across genome increased by 60% for mtDNA but by 30% for nDNA. A high migration rate of animals toward the CEZ would increase both mitochondrial and nuclear diversity, a pattern that does not correspond with our observations. Hence, an increased mutation rate in the CEZ is the most likely explanation for the local genetic novelty and increased genetic diversity for mtDNA and not for nDNA.

### 4.2 A genetic structure consistent with a higher mutation rate in the CEZ

Mitochondrial and nuclear markers differ also in their range of differentiations between populations, but not in the relative structure of these populations. Indeed, based on pairwise *F*_st_ values, the most differentiated populations using mtDNA markers are highly differentiated (>0.4), but not when using nDNA (<0.1). The general structure of these populations is quite similar within the CEZ between mitochondrial and nuclear markers (Figure 4a, b), and for the two types of markers, isolation by distance is not rejected. In amphibians, dispersion is usually male‐biased (reviewed by Helfer et al. (2012), but see Honeycutt et al. (2019)). Since mtDNA is transmitted by females, in case of a strong migration process, there would have been a discrepancy between the relative nuclear and mitochondrial population genetic structure. These results, thus, confirm the absence of a strong tree frog migration process coming from outside the CEZ, and reaffirm the role of mutation processes occurring on mtDNA. The presence of mitochondrial haplotypes exclusive to the CEZ—in contrast to previous studies on bank voles (Wickliffe et al., 2002)—and the absence in the Chernobyl region of haplotypes shared with populations outside the Chernobyl region (except ancestral haplotype), support also the hypothesis of absence of numerous long migration between CEZ and other areas. In this way, the mutation/drift balance explains the higher differentiation found in mtDNA population structure.

### 4.3 Substitution rate and population size at the origin of a “refugia‐like” population

The mitochondrial haplotype network of the CEZ tree frog populations showed a striking structure that differs from what can be expected from the global demographic expansion of the clade D4 (Dufresnes et al., 2016; Stöck et al., 2012). This structure is similar to an ancient diversified population, demographically stable even during the last glacial maximum (Batalha‐Filho et al., 2012; Pulido‐Santacruz et al., 2016). However, it is unlikely that the Chernobyl region would have acted as a refuge zone regarding the global evolutionary history of the *Hyla orientalis* species (Dufresnes et al., 2016) and the possible recent impact just after the 1986 Chernobyl nuclear accident on amphibians (Geras’kin et al., 2008; Vojtovich, 2001). The results of our simulation suggest that a strong mutation rate coupled with populations of small sizes might be responsible for the establishment of the CEZ haplotype network structure. Our haplotype network simulation obtained the observed CEZ haplotype network pattern in 30 years from control local populations identifying two important parameters, a strong nucleotide substitution per haplotype per generation of 0.04 and populations of small effective size inferior to 100 individuals (Figure 6). We noticed a better match between the observed network and the simulated network after 15 generations instead of 10. However, the age of *H*.*orientalis* has been studied in Turkish populations and the majority of breeding females were 3 years old (Altunisik & Özdemir, 2013; Özdemir et al., 2012). Unfortunately, in the case of the CEZ, we have no information about the age of breeding females, but breeding at 3 years would correspond to 10 generations from the accident. We can thus wonder if the CEZ female tree frogs start to breed at 2 years in order to speed up life‐history strategy. A shorter generation time may be an adaptive response to cope with the accumulation of damage in stressful environments (Brans & Meester, 2018; Dutilleul et al., 2017), as those with radioactive contamination.

### 4.4 Can ionizing radiation be at the origin of the increase in substitution rate in the CEZ?

The mitochondrial evolutionary pattern of the CEZ populations, which seems to be the result of a dynamic comparable to an accelerated evolution, is not observed outside the CEZ.

Slavutych's tree frog populations that are geographically close to the CEZ populations do not show the same haplotype structure and did not present any case of heteroplasmy, contrary to the CEZ populations. Knowing the mutagenic ability of ionizing radiation (Breimer, 1988), it seems highly likely that the increase in mitochondrial substitution rate by several hundreds of times compared to the mitochondrial substitution rate normally observed in amphibians has been caused by ionizing radiation. Nevertheless, this study does not allow specifying exactly the relationship between the artificial radionuclide exposure and the evolutionary processes estimated from genetic variations. The positive correlation between mitochondrial nucleotide diversity and ATDRs (currently ranging in the frog samples at the CEZ from 0.007 to 22.4 µGy/h) is in agreement with an effect of ionizing radiation on genetic diversity, but there is no significant correlation between mitochondrial haplotype genetic diversity and ATDRs contrary to the results of Baker et al. on bank voles between haplotype genetic diversity and ambient dose rate (Baker et al., 2017). The ATDR seems to be the most relevant dose rate estimator for a population over a time period, but it does not account for exposure of previous generations that occurred since the accident, even though possible transgenerational effects (Hancock, Vo, Byun et al., 2019; Hancock, Vo, Omar‐Nazir et al., 2019; Sakauchi et al., 2020) and evolutionary processes should be dependent of these historical doses. Because our methodology permitted us to observe only germinal substitutions (the PCR is blind to low‐frequency polymorphism in the DNA sample), or rare cases of somatic substitutions early in the development, these substitutions are not only caused by current exposure to artificial radionuclides, but are also the result of mutations accumulated by individuals exposed to ionizing radiation in previous generations. There is no information on local tree frog population genetics before the accident and, thus, we cannot exclude uncertainties on the determination of the magnitude of the genetic modifications even if the use of Slavutych populations as a proxy of ancestral populations appears consistent. To fully understand the implication of ionizing radiation on the modification of the intensity of evolutionary processes, it should be valuable to compare these results with similar studies conducted in other radio‐contaminated places like the Fukushima prefecture in Japan.

### 4.5 The key role of mitochondrial DNA in evolutionary ecotoxicology

Our results show that the visible higher genetic diversity may not correspond to a classical evolutionary scenario (i.e., an ancestral population) and that mitochondrial markers are useful to assess the mutagenic effect of ionizing radiation (Kam & Banati, 2013). Previous studies (e.g., Fuller et al., 2019) did not find any significant positive correlation between absorbed radiation (ATDRs ranging from 0.064 to 26.4 µGy/h) and nuclear genetic diversity in the freshwater crustacean *Asellus aquaticus* from the Chernobyl region. This study concluded that the exposure to ionizing radiation has not significantly influenced genetic diversity in *A*. *aquaticus* in the Chernobyl area. The analyses of mitochondrial markers might have provided other complementary information pointing toward a mutation process as shown in our study on *H*. *orientalis*. Mitochondrial markers are thus an important tool for estimating the modification intensity of evolutionary processes, but also the probable consequences of mitochondrial mutations on individuals and populations. In humans, mtDNA mutations are responsible for several mitochondrial diseases like optic neuropathy (Johns & Neufeld, 1991), MELAS (Hirano & Pavlakis, 1994), and MERRF syndromes (Shoffner et al., 1990). Because of the possible presence of different mtDNA in a single cell, disease symptoms associated with mtDNA mutation could be generated by quantitative changes in the proportion of mtDNA mutants (Picard et al., 2014). Moreover, at the population level, the maternal transmission of mtDNA can prevent selection against mutations, which are deleterious only when expressed in males (Innocenti et al., 2011) and can lead to a decrease in population viability (Gemmell & Allendorf, 2001).

### 4.6 The necessity of a large space and time scales

Genetic diversity can be sensitive to many environmental parameters (Meeks et al., 2009) and considering a global phylogeographic context could help to overcome this issue. Examining only CEZ and Slavutych tree frog populations would have been insufficient to draw reliable conclusions about evolutionary processes. However, by putting local estimations of genetic diversity of tree frogs (i.e., in the Chernobyl region) in a global phylogeographic context for the species, we were able to get a more accurate picture of the putative effects of radio‐contamination on genetic variations and thus potential evolutionary processes of tree frog populations in the CEZ. Our simulation data show the need for a certain duration of exposure to radiation, as well as the role of other factors like population size, generation time, and the mutation rate, to obtain a network pattern similar to that observed in the CEZ (Figure 6d). It is possible that, depending on the life history of the organisms, genetic effects are different and/or not fully visible. Such difference might explain other recent findings showing an absence of visible radiation‐induced mitochondrial microevolution (Newbold et al., 2019).

## 5 CONCLUSIONS

Our study on the genetics of the Eastern tree frog populations in the CEZ suggests the existence of a strong mutation process on mitochondrial DNA, resulting in an unexpected genetic structure of the CEZ populations comparing to other European populations. One challenge now is to understand the possible consequences of this genotypic effect on population status. Due to the crucial role of mitochondria (Roubicek & Souza‐Pinto, 2017), it seems unlikely that these levels of mutation rate do not result in deleterious effects. The small population size predicted by our simulation may be a consequence of the elimination of nonviable individuals at birth, or due to other deleterious effects of ionizing radiation such as a reduction in breeding success (see e.g., Mappes et al., 2019) or phenotypic disadvantage of mutations (Ballard & Pichaud, 2014; Dowling, 2014). If the effects of these mutations do not fully compromise the maintenance of tree frog populations, it is not necessarily true for other organisms with different life history. With their large clutch sizes (up to 600 eggs per female per year; Broquet et al., 2009), tree frogs seem to be effective at supporting the deleterious effects of mutations, but it might not be the case for organisms with smaller litters for example. More detailed studies on species with different life‐history parameters are clearly needed to have a full picture of the eco‐evolutionary effects of wildlife exposure to radioactive contamination.

## Supporting information

Appendix S1

Appendix S2

## Data Availability

Mitochondrial sequences are available in GenBank, reference number MZ419032–MZ419254 and microsatellites data are available in Figshare at http://doi.org/10.6084/m9.figshare.14828535
