# Supplementary material for: Unusual evolution of tree frog populations in the Chernobyl exclusion zone
Source: Evol Appl. 2022 Jan 26;15(2):203–19. doi: 10.1111/eva.13282 (PMC8867709; doi:10.1111/eva.13282)

Supplemental Information for:

Unusual evolution of tree frog populations in the Chernobyl exclusion zone

Clément Car, André Gilles, Olivier Armant, Pablo Burraco, Karine Beaugelin-Seiller, Sergey Gashchak, Virginie Camilleri, Isabelle Cavalie, Patrick Laloi, Christelle Adam-Guillermin, Germán Orizaola and Jean-Marc Bonzom

Table of Contents:

**Figure S1**	Page 2
**Figure S2**	Page 3
**Table S1**	Page 4
**Table S2**	Page 5
**Table S3**	Page 6
**Table S4**	Page 6
**Table S5**	Page 7
**Table S6**	Page 7
**Table S7**	Page 8
**Figure S3**	Page 9
**Note 1**	Page 10
**Table S8**	Page 11
**Note 2**	Page 12
**Figure S4**	Page 12
**Figure S5**	Page 13
**Figure S6**	Page 14
**Table S9**	Page 15
**Table S10**	Page 16
**Table S11**	Page 16
**Figure S7**	Page 17
**Note 3**	Page 19
**Table S12**	Page 19
**Table S13**	Page 20
**Figure S8**	Page 21
**Figure S9**	Page 22
**Figure S10**	Page 23

Figure S1: Example of heteroplasmy for the individual 12_9 of the A18 population for the 666 nucleotide position for Hyla-L0 and Hyla-H1046 primers.

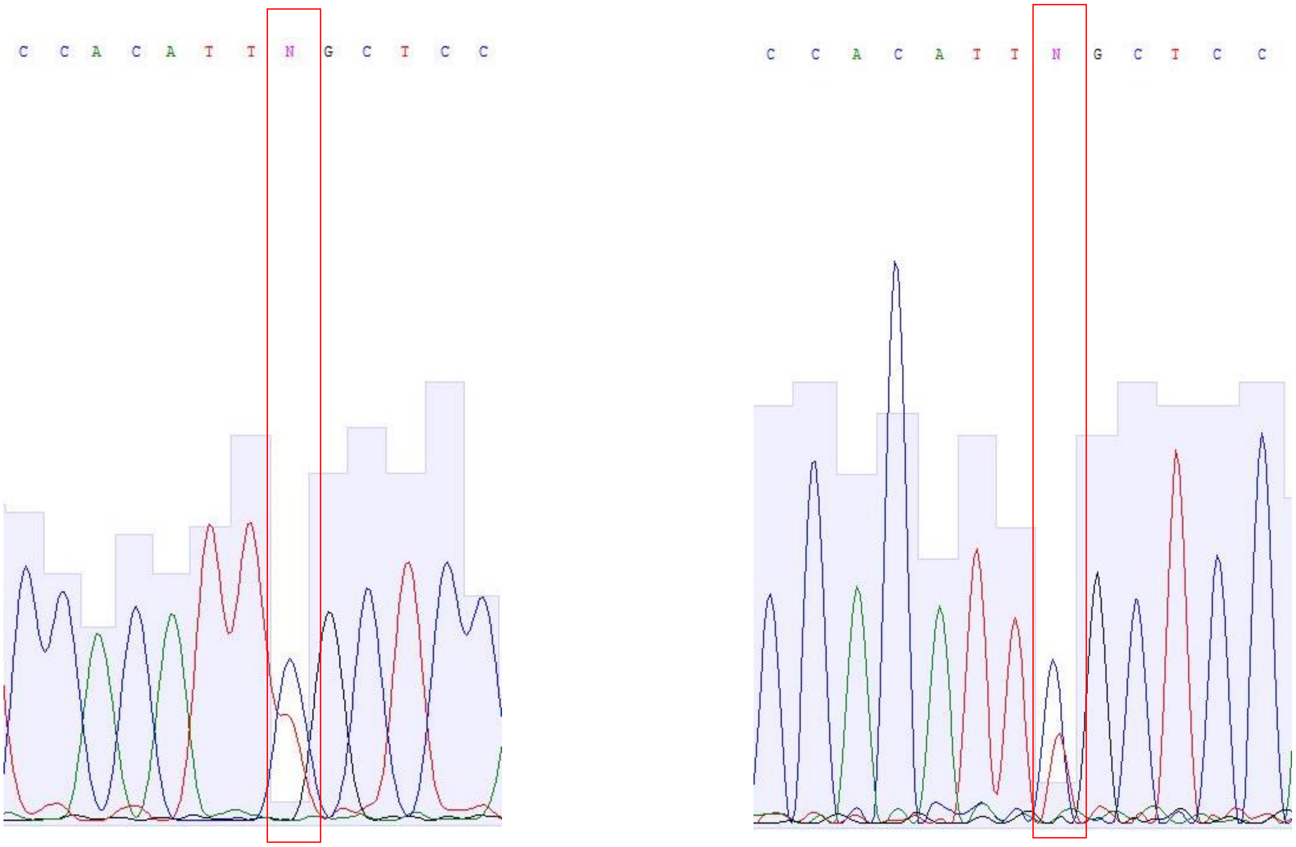

Figure S2: Neighbor-Joining tree separating each year. a,b,c. From cytochrome b. d,e. From microsatellites. a-d. 2016. b-e. 2017. c. 2018

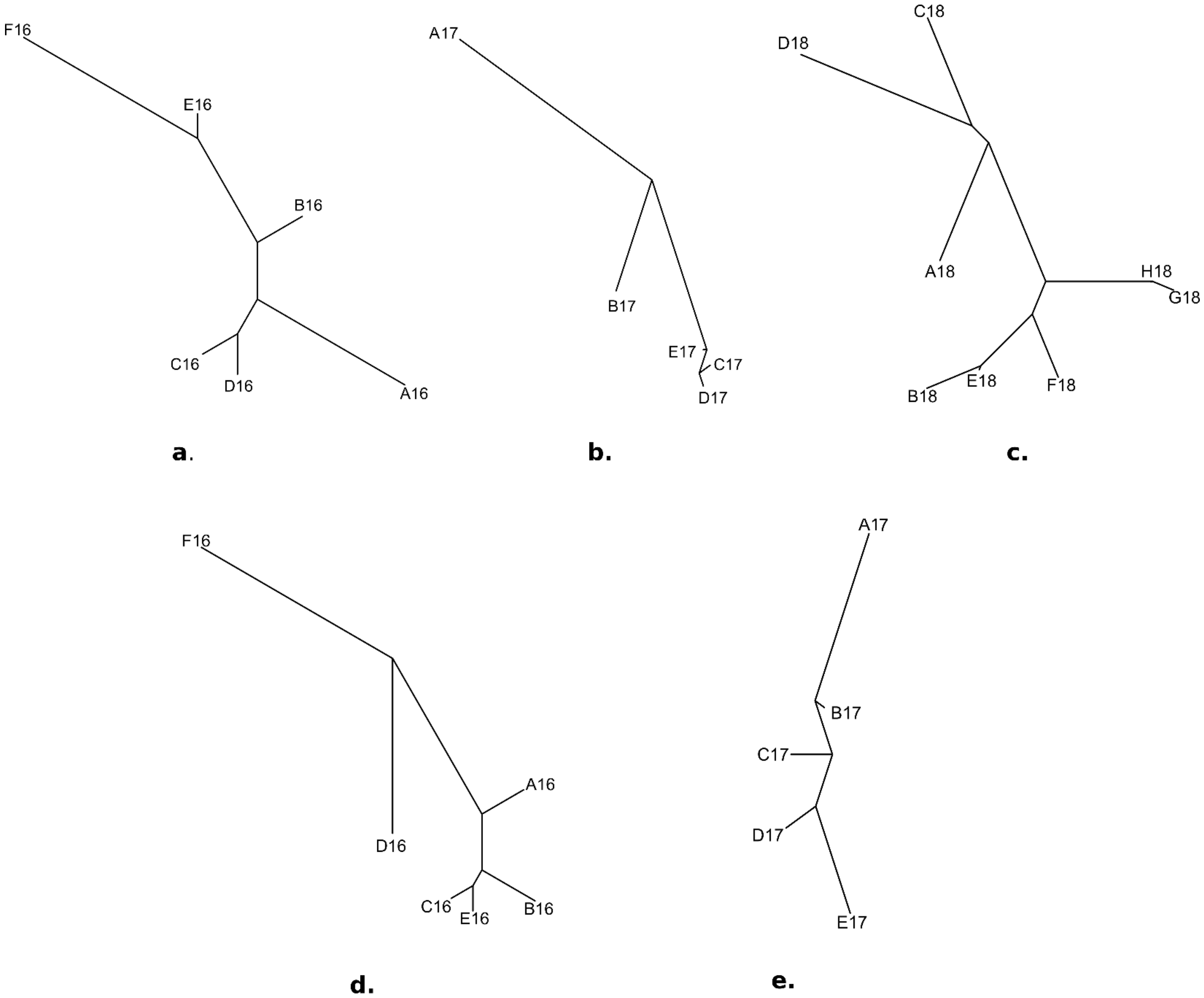

Table S1: Haplotypes found at the Chernobyl exclusion zone (red), Slavutych (green) or both (orange). "." represents the same nucleotide as for the first haplotype for the position considered.

	Nucleotide position																			
Haplotypes	57	78	92	96	105	151	153	199	259	279	322	426	427	594	666	690	806	874	934	939
1	T	T	C	C	C	G	C	C	A	A	G	T	A	G	C	C	T	C	A	A
2	.	C	.	.	.	A	.	.	.	.	.	.	.	.	.	.	.	T	G	.
3	.	.	.	.	.	.	T	.	.	.	.	.	.	.	.	.	.	.	.	.
4	.	C	.	.	.	.	.	.	.	T	.	.	.	.	.	.	.	.	.	.
5	.	C	.	.	.	.	.	T	.	.	.	.	.	.	T	.	.	.	.	.
6	.	C	.	.	.	.	.	.	.	.	.	.	.	.	.	.	.	.	.	.
7	.	.	.	.	.	.	.	.	.	.	.	C	.	.	.	.	.	.	.	.
8	.	C	.	T	.	.	.	.	.	.	.	.	.	.	.	.	.	.	.	.
9	.	C	.	.	.	.	.	.	.	.	.	.	.	.	.	T	.	.	.	.
10	.	C	.	.	.	.	.	.	.	.	.	.	.	.	T	.	.	.	.	.
11	.	C	T	.	.	.	.	.	G	.	.	.	.	.	.	.	.	.	.	G
12	.	C	T	.	.	.	.	.	G	.	.	.	.	.	.	.	.	.	.	.
13	.	C	.	.	.	.	.	.	.	.	.	.	G	.	.	.	.	.	.	.
14	.	C	.	.	T	.	.	.	.	T	.	.	.	.	.	.	.	.	.	.
15	C	C	.	.	.	.	.	.	.	.	.	.	.	.	.	T	.	.	.	.
16	.	C	.	.	.	.	.	T	.	.	.	.	.	.	.	.	.	.	.	.
17	.	C	.	.	.	.	.	T	.	.	.	.	.	.	T	.	C	.	.	.
18	.	C	.	.	.	.	.	.	.	T	A	.	.	.	.	.	.	.	.	.
19	.	C	.	.		.	.	.	.	.	.	.	.	A	.	.	.	.	.	.

Table S2: Mutations measured in Chernobyl exclusion zone (red) and Slavutych (green) or both (orange) Eastern tree frogs on the 1st, 2nd or 3rd codon position, and modifications of amino acids.

Position	1st	2nd	3rd	AA
57			C/T	
78			C/T	
92		C/T		Ala/Val
96			C/T	
105			C/T	
151	A/G			Val/Ile
153			C/T	
199	C/T			
259	A/G			Met/Val
279			A/T	
322	A/G			Val/Ile
426			C/T	
427	A/G			Thr/Ala
594			G/A	
666			C/T	
690			C/T	
806		T/C		Ile/Thr
874	C/T			Leu/Phe
934	A/G			Thr/Ala
939			A/G	

Table S3: Populations and substitutions for the 7 tree frogs with heteroplasmy.

Nucleotide position	Population	Number of individuals	Year of sampling
153 – C/T	AB17	2	2017
199 – C/T	E17	1	2017
322 – C/T	F18	3	2018
666 – C/T	A18	1	2018

Table S4: Statistical tests on different estimates of genetic diversity. Comparison of the genetic diversity of Chernobyl exclusion zone (CEZ) Eastern tree frog populations with other European populations (non-parametric Mann-Whitney/Wilcoxon tests for cytochrome b).

Diversity index	W statistic	p.value	Median CEZ	Median other European populations
h	91	0.005 **	0.7308	0.6071
π	99	0.0004 ***	0.0024	0.0008
θ_S	96	0.001 ***	1.9868	0.8163
θ_K	92	0.006 **	2.5006	1.2535
θ_π	99	0.0004 ***	2.183	0.7556
nrH	89	0.011 *	3.140	2.520

Table S5: Comparison of the genetic diversity of Chernobyl exclusion zone (CEZ) Eastern tree frog populations with other European populations (non-parametric Mann-Whitney/Wilcoxon tests for microsatellites markers).

Diversity index	W statistic	p.value	Median CEZ	Median other European populations
He	13	0.2398	0.2064	0.2400
Ho	17	0.5185	0.1887	0.2250
Fis	25	0.7972	0.0920	0.0670
Gene diversity	13	0.2398	0.2135	0.2410

Table S6: Correlation tests between the genetic diversity of Eastern tree frog populations in the Chernobyl region, i.e. all the populations of Chernobyl exclusion zone and the two populations of Slavutych and the average total dose rates (ATDRs) of ionizing radiation absorbed by each population total (non-parametric Spearman rank correlation for cytochrome b).

Diversity index	S statistic	rho	p.value
h	658	0.1936	0.455
π	294	0.6397	0.007 **
θ_S	536	0.343	0.1776
θ_K	581.86	0.287	0.264
θ_π	294	0.6397	0.007 **
nrH	634.78	0.222	0.3916

Table S7: Non-parametric Spearman rank correlation for microsatellites markers between the genetic diversity of Eastern tree frog populations in the Chernobyl region, i.e. all the populations of Chernobyl exclusion zone and the two populations of Slavutych and the average total dose rates (ATDRs) of ionizing radiation absorbed by each population.

Diversity index	S statistic	rho	p.value
He	194	-0.617	0.08573
Ho	148	-0.233	0.6617
Fis	176	-0.467	0.2125
Gene diversity	194	-0.617	0.08573
AR	194.31	-0.619	0.07535
PA	221.13	-0.8427	0.004 **

Figure S3: Genetic diversity for the more polymorphic microsatellite loci on the Averaged Total Dose Rate (ATDR) ($\mu Gy.h^{-1}$) for each population in the Chernobyl region. There is no relation for any of these markers (spearman's rank correlation tests).

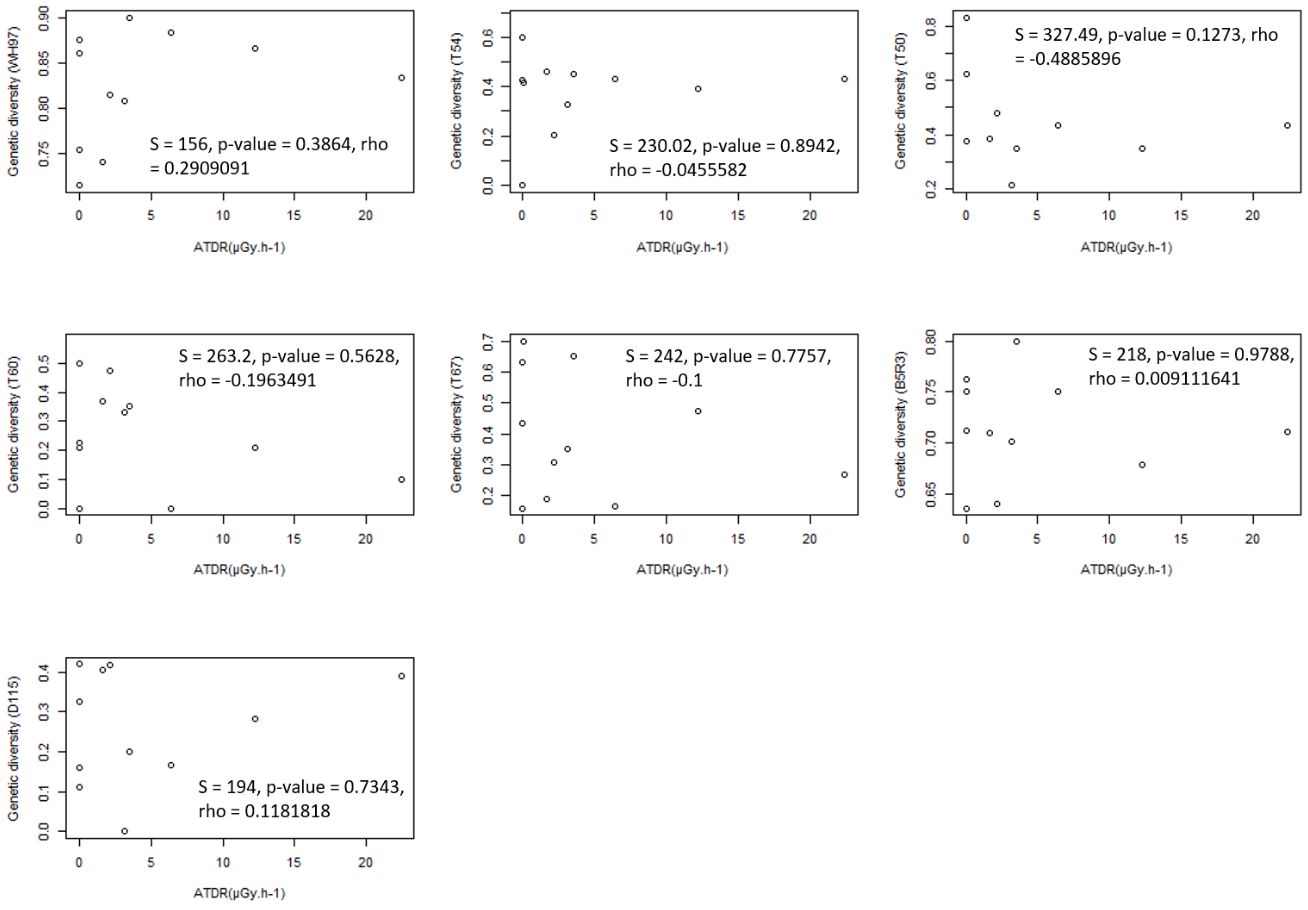

Note 1: Amplification protocols

To sequence the cytochrome b coding gene, a PCR amplification was performed using Hyla-L0: ATGGCCCCTGTTTTACGCAA et Hyla-H1046: TAAATGGGTCTTCTACTGG primers (Dufresnes et al., 2016; Stöck et al., 2008). The amplification protocol was for 25µl of final PCR volume: 2.5µl of Qiagen PCR buffer (with 1.5mM of MgCl2), 0.5µl of dNTPs (10mM), 1.5µl of each primer (10µM), 0.1µl of Qiagen Taq (5 units/µl), and 3µl of DNA (10ng/µl), 15.9 µl H_2O. PCR cycles: 1 cycle at 94°C for 1'; followed by 38 cycles 94°C for 30", 48°C for 1', 72°C for 1'; and 1 cycle at 72°C for 10'.

Dufresnes, C., Litvinchuk, S. N., Leuenberger, J., Ghali, K., Zinenko, O., Stöck, M., & Perrin, N. (2016). Evolutionary melting pots : A biodiversity hotspot shaped by ring diversifications around the Black Sea in the Eastern tree frog (Hyla orientalis). Molecular Ecology, 25(17), 4285-4300.

Stöck, M., Dubey, S., Klütsch, C., Litvinchuk, S. N., Scheidt, U., & Perrin, N. (2008). Mitochondrial and nuclear phylogeny of circum-Mediterranean tree frogs from the Hyla arborea group. Molecular Phylogenetics and Evolution, 49(3), 1019-1024.

For microsatellites markers, four multiplex amplifications were performed for the 21 microsatellite markers (Dufresnes et al., 2014; Table S8). The amplification protocol was for 10µl of final PCR volume: 5µl of Qiagen Multiplex Master Mix, 1µl of each primer mix, 1µl of H_2O and 3µl of DNA (10ng/µl). PCR cycles: 1 cycle at 95°C for 15'; followed by 35 cycles at 94°C for 30", 58°C for 1'30", 72°C for 1'; and 1 cycle at 60°C for 30'.

Dufresnes, C., Brelsford, A., Béziers, P., & Perrin, N. (2014). Stronger transferability but lower variability in transcriptomic- than in anonymous microsatellites: evidence from Hylid frogs. Molecular Ecology Resources, 14(4), 716–725.

Table S8: Nuclear microsatellites used for genetic analysis.

Primer (repeated pattern)	Size range	Multiplex	Concentration (µM)	Forward primer	Reverse primer	Nb alleles
Ha-T50 (CCG)7(CCA)1(CCT)5	129 - 144	H	0.10	F: *HEX*-CAGCCCAACTGACTGGTTTT	R: GGGGAAGACTTTGACCCTCA	6
Ha-T53 (TT)1(TC)5	357 - 364	H	0.20	F: *HEX*-TCTCCTGTCCTTCACCCAAC	R: CTTCCCAGCCTGGAACATC	2
Ha-T54 (AT)6	200 - 210	H	0.10	F: *FAM*-GTGTGTAGGACCCAGGGAGA	R: TTGCTTCCGCTTGTGTAGTG	8
Ha-T55 (AG)7	296 - 314	H	0.10	F: *FAM*-ATGGAAGGCTGAAGAGAGCA	R: CCAAAGGGTTAAATGCAGGA	3
Ha-T56 (AT)7	239 - 244	H	0.20	F: *ATTO*-TGCAAAAATGCCATGAAGTC	R: TTTGGAGACATCACGGTTGA	5
Ha-T58 (TCC)4…(TCA)6…(CTA)4 CTG(TCA)4	234 - 243	H	0.10	F: *HEX*-TCCCGAAAGGACTACTGCTG	R: ACGCACAGGAGGAGAAAGAA	4
Ha-T60 (CAA)5(CAG)3	271 – 286	I	0.10	F: *FAM*-ATTGCGAAAAACTGGTGGTT	R: GCTTTTCCCAGATCAACAGG	6
Ha-T61 (CAG)5	228 – 236	I	0.25	F: *HEX*-CCGCAAAGATAATCCCAATC	R: AGGCTGCTGCATTAGATGGT	2
Ha-T63 (TTC)5	207	I	0.10	F: *ATTO*-TTCTGACCTCTCGGTTTGCT	R: ATGTAAAGGCGCTGATGGAG	1
Ha-T66 (CACACAT)1(TG)7	106	I	0.10	F: *FAM*-CTCTTTCGGGTTCCATGCT	R: TCCATTGTGCTGATCGTGTT	1
Ha-T67 (CAT)6	265 – 289	I	0.25	F: *ATTO*-GGGCAGCTTTATTTTTCAGC	R: AGTGGCACCTCCAATAAAGG	6
Ha-T68 (CA)7	355 – 362	I	0.10	F: *HEX*-AGGGCAGAGATACAGGCGTA	R: TGAAACAAATACCGGACTGC	5
Ha-A11 (CA)14	193 - 202	C	0.22	F: *ATTO*-CCTCCCTCACTCTGCTGAC	R: CAATCCCCGAAAAACATTG	3
Ha-A127 (TG)14	293 – 295	C	0.30	F: *HEX*-CTCTGGGTTGCACTACTTAGTC	R: TTCAGGGCTAATTCTTTGTATG	2
Ha-B5R3 (TC)13	346 – 363	C	0.05	F: *FAM*-CCCCTTTAGAGTCGCCATAC	R: AGCCATCTTGTGGTCAGTCA	6
WHA1-67 (CA)21	204 – 226	C	0.29	F: *ATTO*-GCTTTACACATGGGGGTAT	R: CACTCCTTTTAGAGTATGTTGTTG	12
Ha-D104 (TAGA)7	212 – 221	D	0.18	F: *FAM*-GCTGGCTGACTTATTCTTTG	R: TCTTCTCTCCACGGTCTTC	4
Ha-D115 (TAGA)16	198 – 210	D	0.30	F: *FAM*-GTTTTTCGATTCCTGGATAAC	R: TGGGAGTTTTCAAAAGTGAC	6
Ha-E2 (CAA)7	142 – 152	D	0.30	F: *HEX*-ACAACTTCCAACTGGAGTCAAC	R: CCTTAGTGGGAGCTGTAATCAC	3
Ha-A110 (CA)15	200 – 207	D	0.30	F: *ATTO*-AAGGGTTAAATCACCTATCC	R: ACGCAAAAAACATCTGTG	4
Ha-A119 (CT)14(CA)6TA(CA)14	220 - 224	D	0.15	F: *ATTO*-CAACTTCCCCCTCTGTTC	R: GCTGAGTGTGAGTGTGTTTG	2

Note 2: Total dose rate calculation

Figure S4: Exposure scenarios applied to estimate the Dose Coefficients (illustration modified from Giraudeau et al, 2018[1]). Because of the characteristics of the Chernobyl exclusion zone compared to the Fukushima situation, the depth of the different microhabitats and the time of exposure to the four scenarios has been modified.

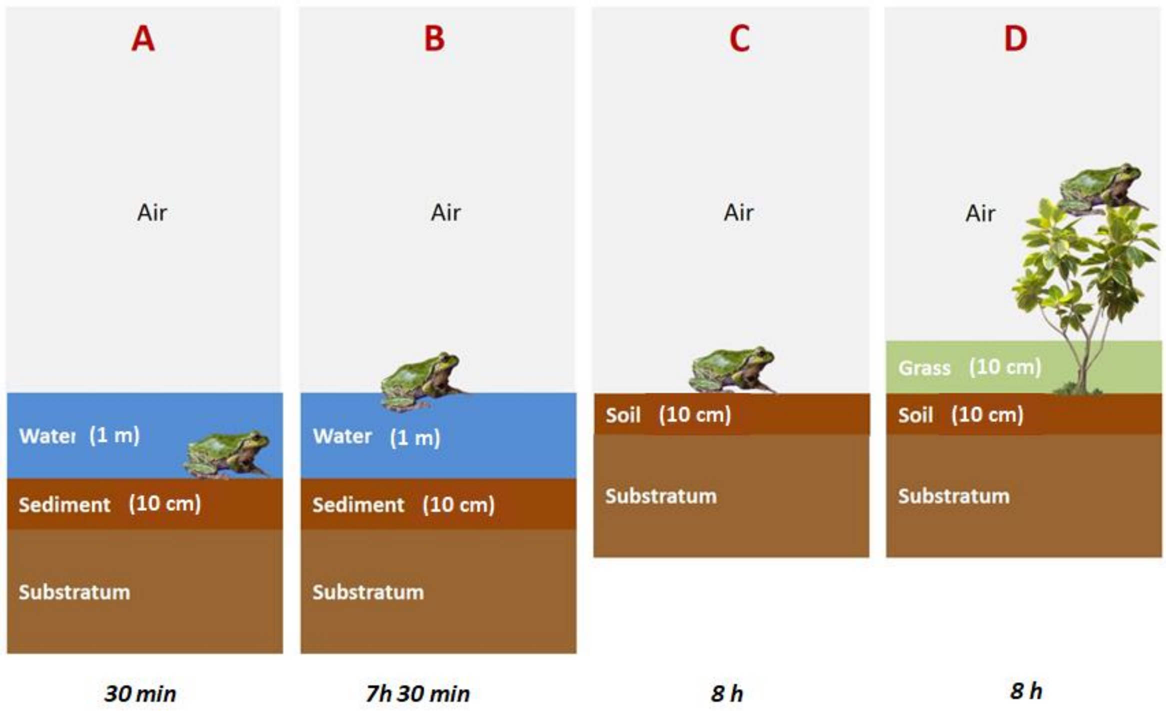

Figure S5: Dose distribution within and between populations. Representation of total dose rates absorbed by individual (TDRs) ($\mu Gy.h^{-1}$) for the Eastern tree frog populations of the Chernobyl exclusion zone. Colours represent the ambient dose rate gradient measured on the field (>10 $\mu Gy.h^{-1}$, dark red; >5 $\mu Gy.h^{-1}$, red, >3 $\mu Gy.h^{-1}$, orange, >2 $\mu Gy.h^{-1}$, dark yellow; >1 $\mu Gy.h^{-1}$, yellow; <1 $\mu Gy.h^{-1}$, blue).

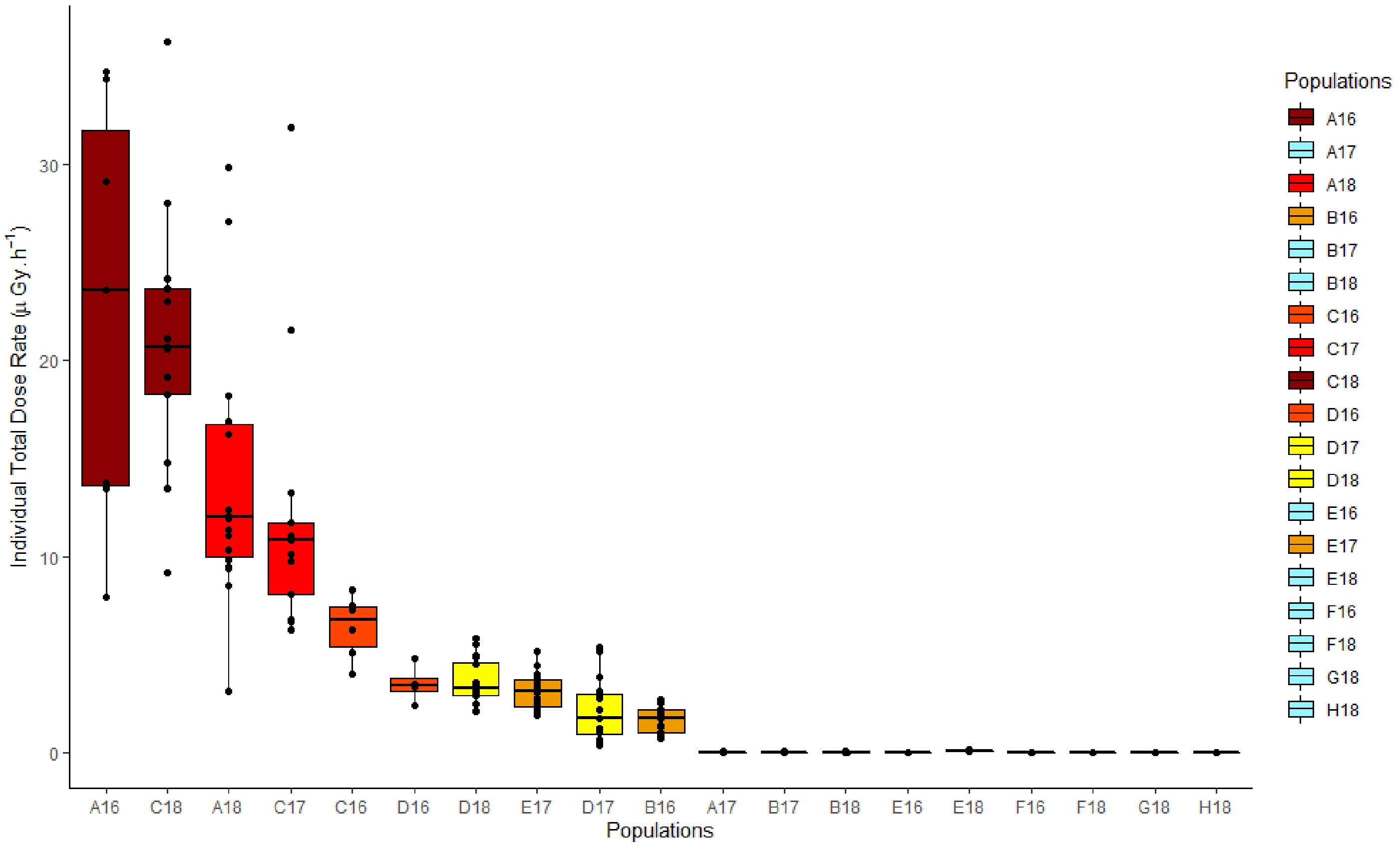

Figure S6: Representation of the internal (blue) and external (pink) average total dose rates (ATDRs) of ionizing radiation absorbed (µGy.h^{-1}) for Chernobyl exclusion zone populations rate.

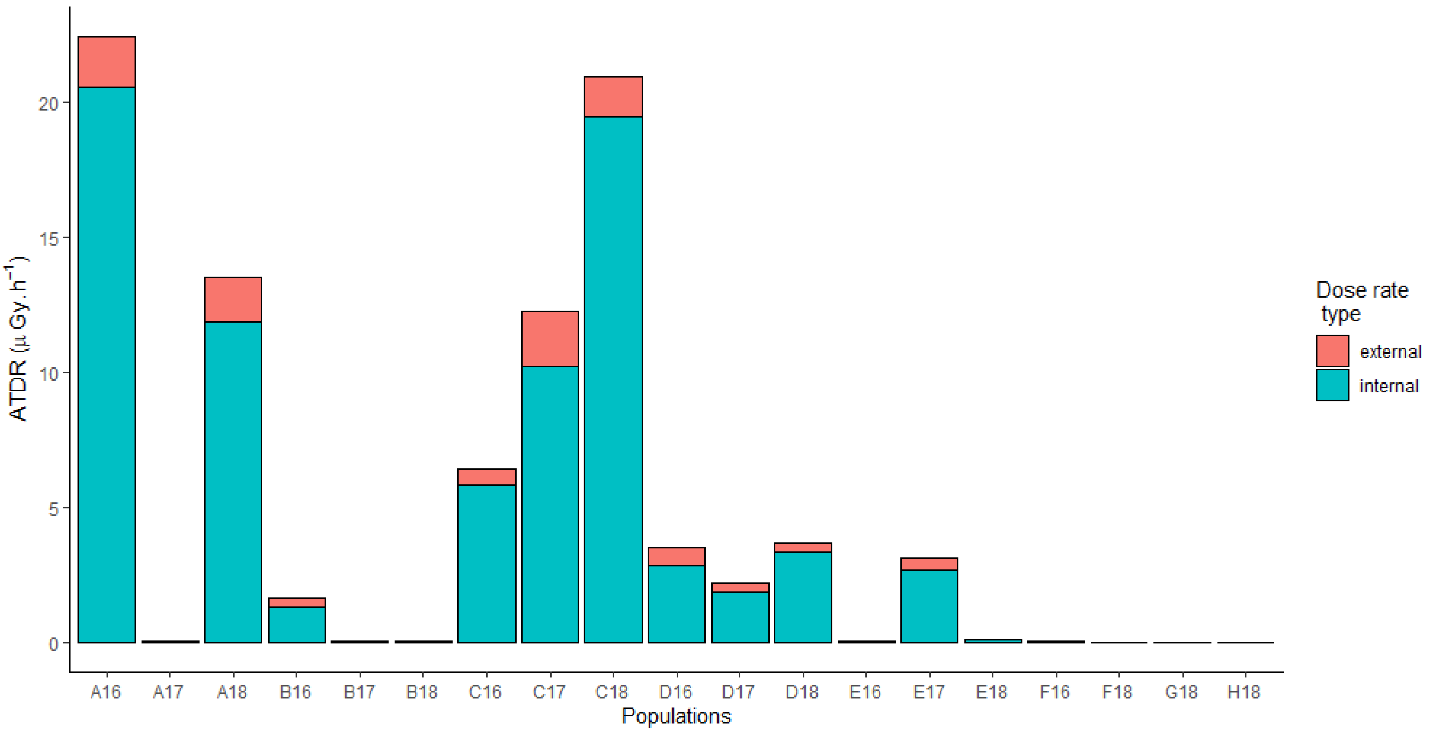

In order to assess the contribution of rare radionuclides compared to ^{137}Cs and ^{90}Sr on the total dose rate, dose rate relative to each radionuclide was reconstructed. A set of realistic extreme activity concentrations in soils estimated for February 2009 for every radionuclide was used[2–4]. The radioactive decay was applied to each minimum and maximum value.

Table S9: Minimal and maximal soil activity concentration (Bq.kg-1) in 2017 for radionuclides present in the Chernobyl exclusion zone.

	Soil activity concentration (Bq.kg^{-1})	
	Minimum	Maximum
^{137}Cs	56.3	395942
^{90}Sr	29.7	368695
^{241}Am	0.67	12192
^{238}Pu	0.76	3435
^{239}Pu	2.43	8761
^{240}Pu	2.43	8753
^{234}U	0.95	20.0
^{238}U	1.07	11.0
^{60}Co	0.04	196
^{154}Eu	0.51	1130

The internal activity concentration was reconstructed using concentration ratio estimated by Beresford et al.[5] on *Rana arvalis* and ERICA 1.3 [6] for other non-documented radionuclides.

Table S10: Minimal and maximal frog activity concentration ($Bq.kg^{-1}$) in 2017 for radionuclides present in the Chernobyl exclusion zone.

	Frog activity concentration ($Bq.kg^{-1}$)	
	Minimum	Maximum
^{137}Cs	21.88	154021
^{90}Sr	19.03	235964
^{241}Am	0.00	10.75
^{238}Pu	0.02	88.28
^{239}Pu	0.06	225.16
^{240}Pu	0.06	224.96
^{234}U	0.00	0.10
^{238}U	0.01	0.05
^{60}Co	0.01	37.39
^{154}Eu	0.02	38.44

Dose coefficient for each radionuclide/scenario was calculated with EDEN software 7. Similarly to the total dose rate calculation presented on Material and Methods, external and internal dose rate were compiled to estimate the total dose rate for minimal and maximal soil activity concentration.

Table S11: Minimal and maximal Total dose rate ($\mu Gy.h^{-1}$) for radionuclides present in the Chernobyl exclusion zone.

	Total dose rate	
	Minimum	Maximum
^{137}Cs	0.003	20.4
^{90}Sr	0.007	80.3
^{241}Am	0.00002	0.42
^{238}Pu	0.0006	2.56
^{239}Pu	0.002	6.12
^{240}Pu	0.002	6.13
^{234}U	0.0001	0.003
^{238}U	0.0001	0.001
^{60}Co	0.000004	0.02
^{154}Eu	0.00002	0.05

Figure S7: Distribution of radionuclide dose rate among total dose rate for minimal and maximal soil activity concentration. 90Sr and 137Cs are the principal contributors of total dose rate (respectively 69% and 87%).

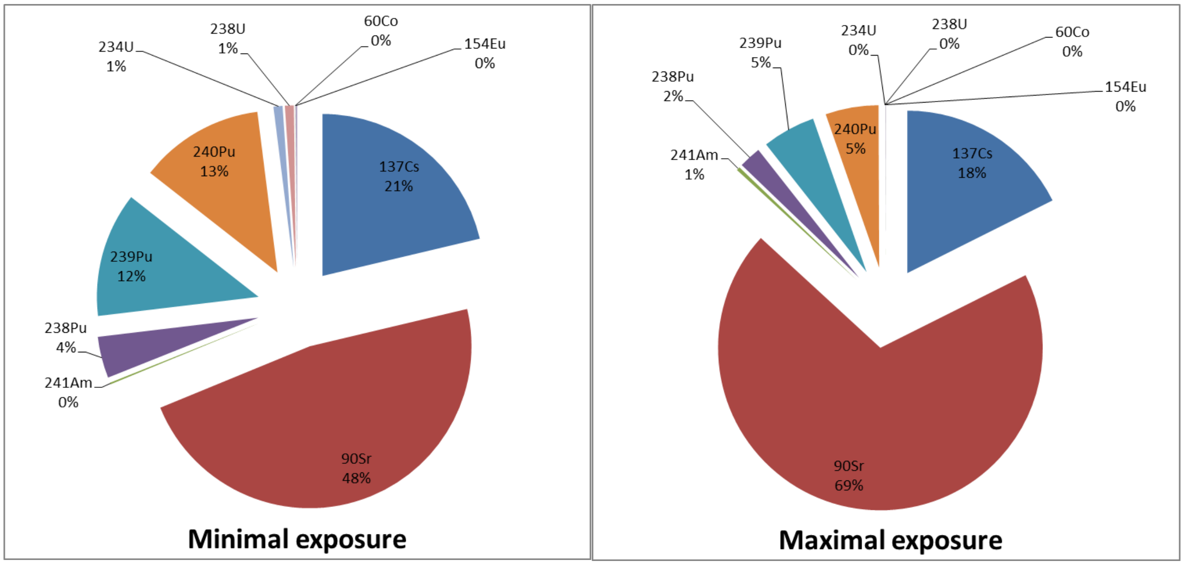

1. Giraudeau, M. et al. Carotenoid distribution in wild Japanese tree frogs (*Hyla japonica*) exposed to ionizing radiation in Fukushima. *Sci. Rep.* 8, 1–11 (2018).
2. Theodorakopoulos, N. Analyse de la biodiversité bactérienne d'un sol contaminé de la zone d'exclusion de Tchernobyl et caractérisation de l'interaction engagée par une souche de Microbacterium avec l'uranium. (Université d'Aix-Marseille, 2013).
3. Chapon, V. et al. Microbial diversity in contaminated soils along the T22 trench of the Chernobyl experimental platform. *Appl. Geochem.* 27, 1375–1383 (2012).
4. Lecomte-Pradines, C. et al. Soil nematode assemblages as bioindicators of radiation impact in the Chernobyl Exclusion Zone. *Sci. Total Environ.* 490, 161–170 (2014).
5. Beresford, N. A. et al. Radionuclide transfer to wildlife at a 'Reference site' in the Chernobyl Exclusion Zone and resultant radiation exposures. *J. Environ. Radioact*. 211, 105661 (2020).

6. Brown, J. E. et al. The ERICA Tool. *J. Environ. Radioact*. 99, 1371–1383 (2008).
7. Beaugelin-Seiller, K., Jasserand, F., Garnier-Laplace, J. & Gariel, J. C. Modeling radiological dose in non-human species: principles, computerization, and application. *Health Phys*. 90, 485–493 (2006).

Note 3: Mitochondrial haplotype network simulations

Table S12: Prior parameters of the first simulation part. These parameters are chosen to be representative of a classical frog population in a wild environment.

Founder population size (N_0)	N_0 = 500
Frequencies of haplotypes in the founder population	Two populations were sampled in Slavutych and may represent the closest local control population. The two set of haplotype frequencies were chosen as two modalities for this parameter. G18 (4 haplotypes): f1 = 0.6, f2 = 0.2, f3 = 0.1, f4 = 0.1 and H18 (2 haplotypes): f1 = 0.95, f2 = 0.05, f3 = 0.0, f4 = 0.0. Each haplotype was separated by one mutation.
Population size for each generation (N_{1-n})	For each generation, N_{min} and N_{max} were sampled in a uniform distribution U(1000-5000) corresponding to an increase of the population size for the first generation N_1 and then a fluctuating population size in the CEZ (balance between the number of dead and alive specimens).
Generation time	Because of the 30 years separating us from the Chernobyl nuclear power plant accident, a number 15 and 10 tree frog generations was chosen, considering a generation time of respectively 2 and 3 years[1,2].
Nucleotide substitution rate μ	A classical rate of nucleotide substitution in mitochondrial DNA for amphibian equal to 20.37×10^{-9} substitution/nucleotide/year (in Lynch 2007 p. 320[1]) was chosen. Considering that one generation for amphibian equal to one year according to Lynch 2007 and considering the 950 bases of the cytochrome b gene we obtain a nucleotide substitution rate μ of 1.94×10^{-4} substitution/haplotype/year (≈ 0.0002).

Table S13: Prior parameters of the second simulation part. These parameters are chosen considering the results of the first simulation part: the first parameters being not able to obtain the diversity of populations of Chernobyl exclusion zone, population sizes should be smaller and nucleotide substitution rate should be higher.

Founder population size (N_0)	Three modalities: N_0 = 100, 250 or 500.
Frequencies of haplotypes in the founder population	As for the first simulation part, the two set of haplotype frequencies of Slavutych populations were chosen as two modalities for this parameter. G18 (4 haplotypes): f1 = 0.6, f2 = 0.2, f3 = 0.1, f4 = 0.1 and H18 (2 haplotypes): f1 = 0.95, f2 = 0.05, f3 = 0.0, f4 = 0.0. Each haplotype was separated by one mutation.
Population size for each year ($N_{1\text{-}30}$)	Three modalities: for each year, N_{min} and N_{max} were sampled in a uniform distribution U(50-100), U(100-200) or U(200-300).
Generation time	Because of the 30 years separating us from the Chernobyl nuclear power plant accident, a number of 15 and 10 tree frog generations was chosen, considering a generation time of respectively 2 and 3 years[1,2]
Nucleotide substitution rate μ	Six modalities: 0.005, 0.01, 0.02, 0.04, 0.06, 0.08 using an infinite site model.

1. Altunisik, A. & Özdemir, N. Body size and age structure of a highland population of *Hyla orientalis* Bedriaga, 1890, in northern Turkey. *Herpetozoa* 26, 49–55 (2013).
2. Özdemir, N. et al. Variation in body size and age structure among three Turkish populations of the treefrog *Hyla arborea*. *Amphibia-Reptilia* 33, 25–35 (2012).
3. Lynch, M. & Walsh, B. *The origins of genome architecture*. vol. 98 (2007).

Figure S8: Distribution of the five haplotype network descriptive statistics for the five percentile closest simulated values. The simulated median (red) and mean (blue) present huge differences from the observed (green, Table S4).

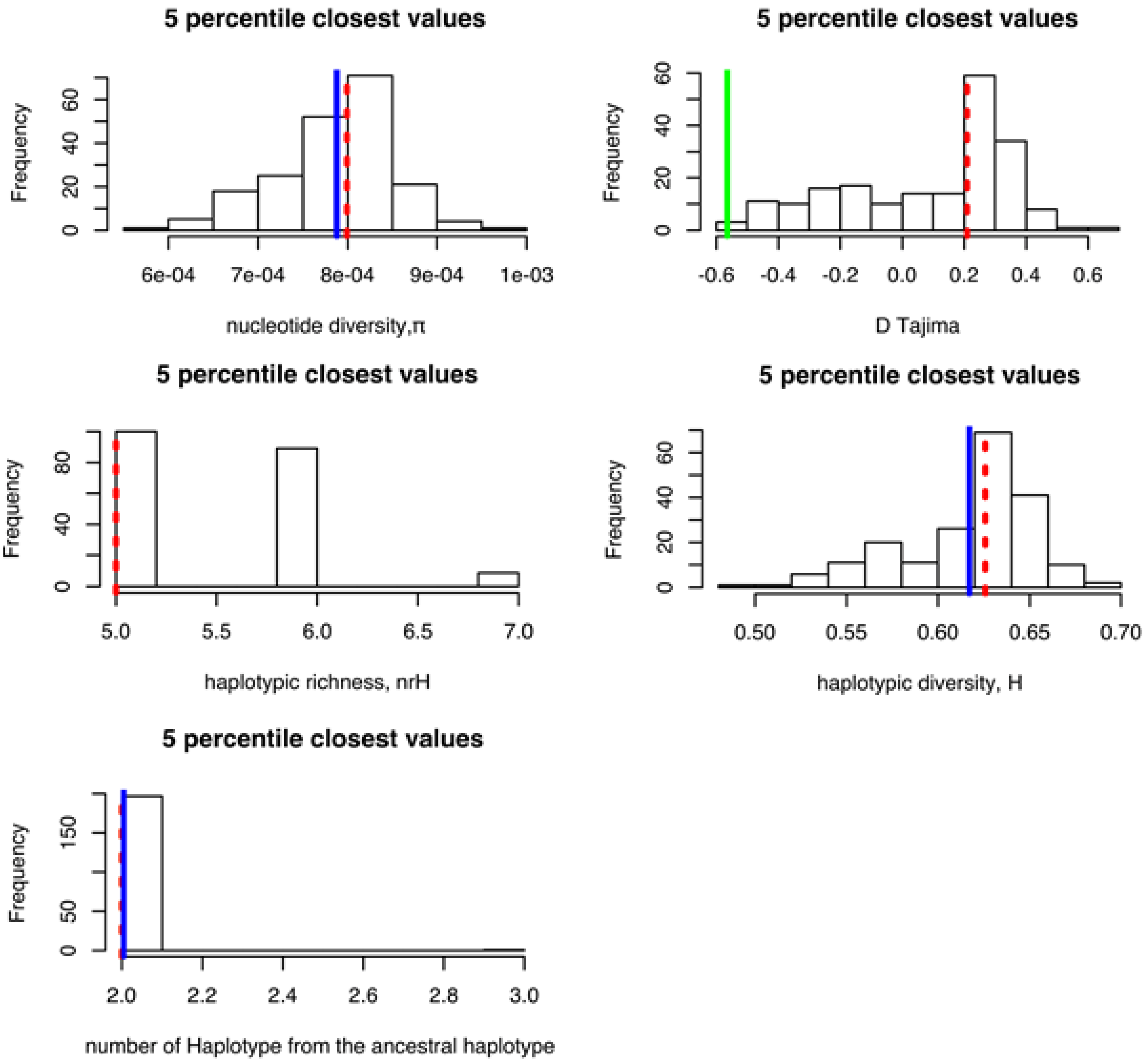

Figure S9: Distribution of the five haplotype network descriptive statistics for the five percentile closest simulated values. The simulated median (red) and mean (blue) are close to the observed ones (green = mean, Table S4).

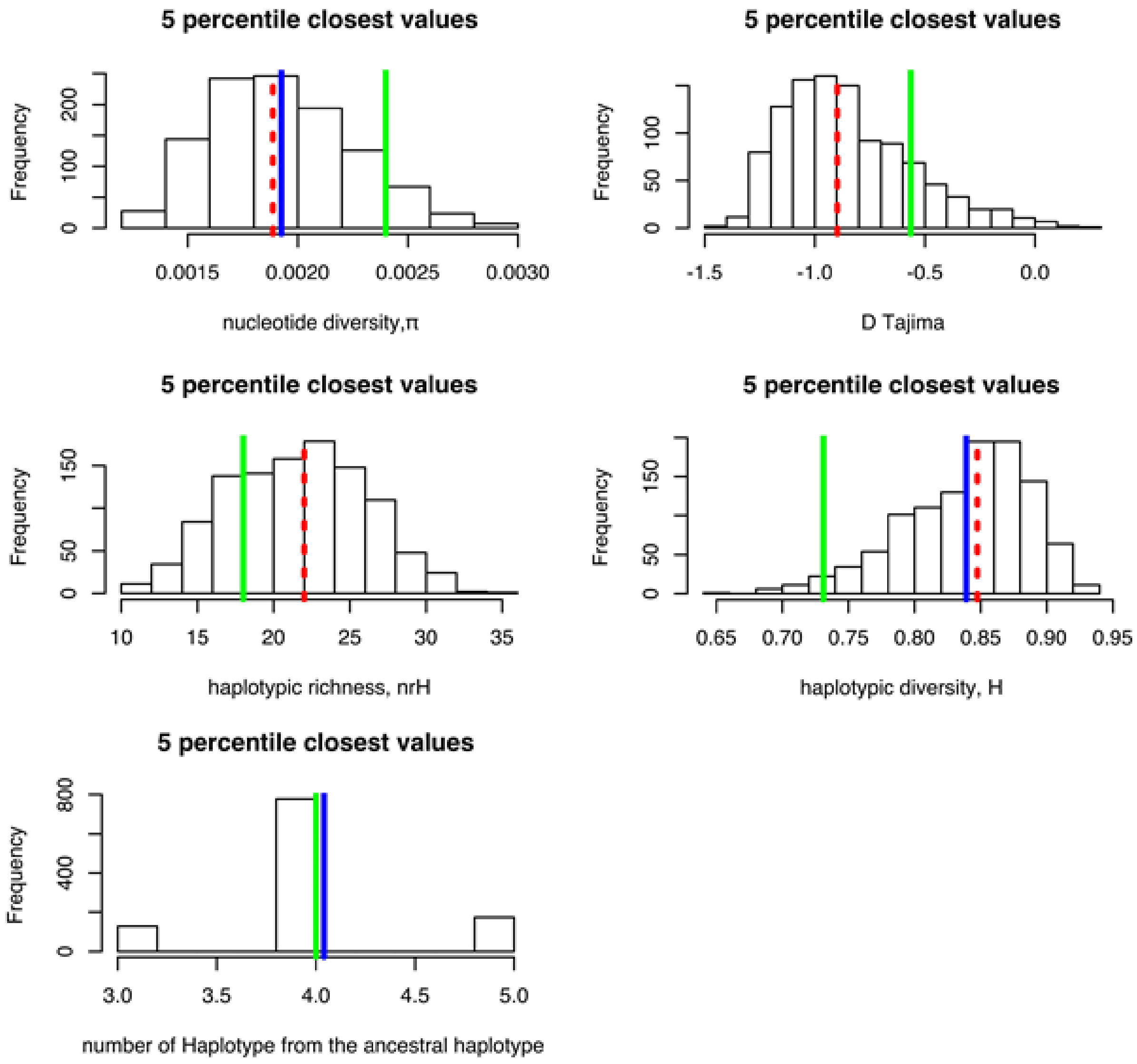

Figure S10: Distribution of the values of parameters μ, N_{max}, N_0, number of generations and haplotype frequencies of the founder population for the 5 percentile closest haplotype networks (red = median, blue = mean).

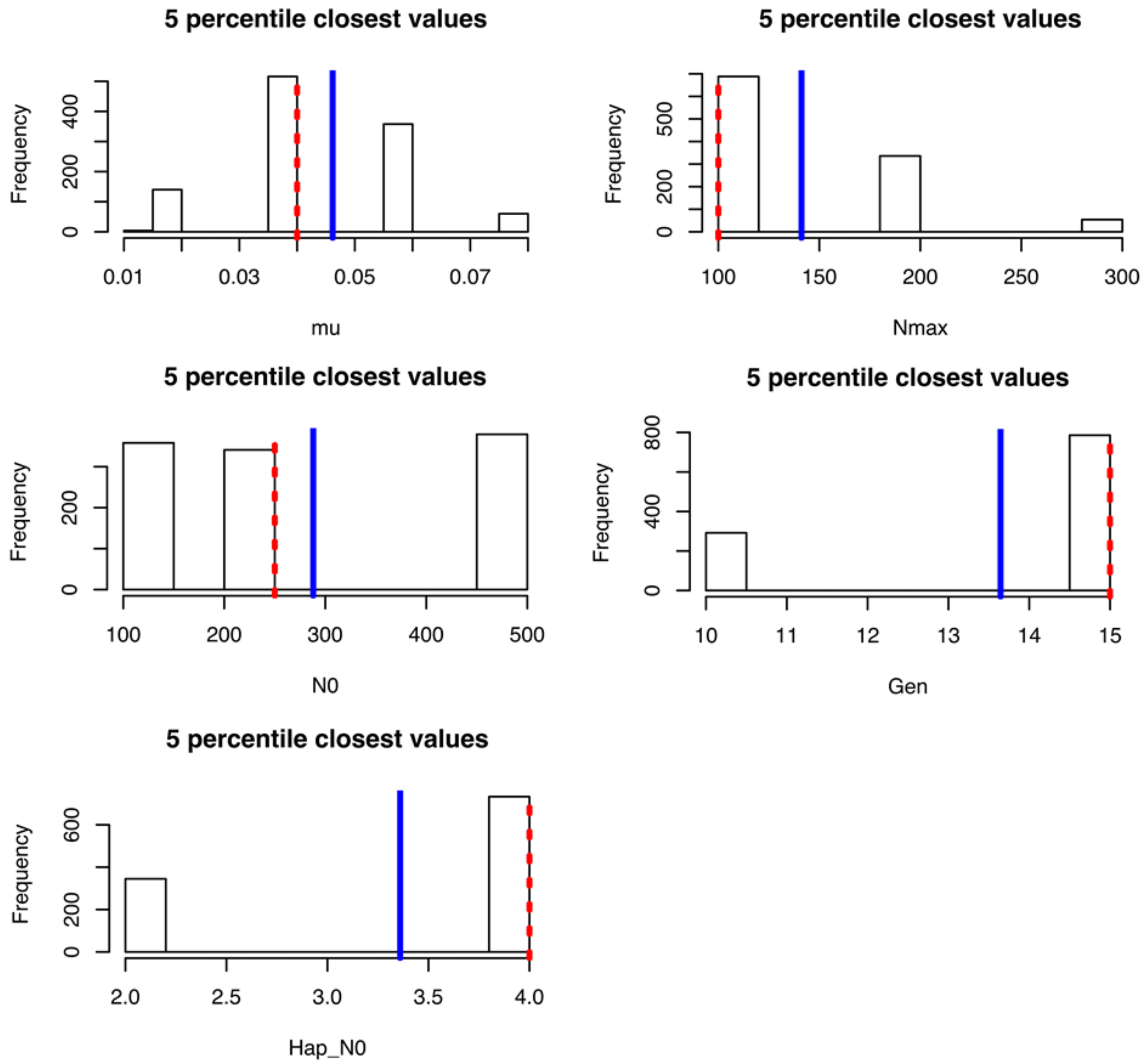

Supplement: Supplementary file 1 — Appendix S1 [file EVA-15-203-s002.pdf]
